# Functional trait syndromes structure macrophyte diversity and functional redundancy across flowing and impounded freshwater systems

**DOI:** 10.3389/fpls.2026.1815637

**Published:** 2026-04-21

**Authors:** Dragana Vukov, Mirjana Ćuk, Miloš Ilić

**Affiliations:** Department of Biology and Ecology, Faculty of Sciences, University of Novi Sad, Novi Sad, Serbia

**Keywords:** aquatic macrophytes, community assembly, ecological stability, functional diversity, functional redundancy, hydrological regime, trait syndromes

## Abstract

Trait-based approaches provide a powerful framework for understanding community assembly and ecosystem stability in freshwater ecosystems, yet functional responses of aquatic macrophytes to hydrological regime and environmental gradients remain incompletely resolved. We analyzed macrophyte communities across flowing (rivers and artificial canals) and impounded (lakes and reservoirs) freshwater systems in Serbia using an explicitly audited functional trait matrix. Multivariate ordination of species traits revealed a structured functional space organized along two principal gradients: (i) a growth-form and mechanical axis contrasting emergent, rigid species reproducing predominantly by seeds with submerged, flexible species relying mainly on vegetative propagation, and (ii) a persistence axis separating annual from perennial strategies. Projection of site-level communities into this trait space indicated significant but partial functional differentiation between hydrological types, with substantial overlap between flowing and impounded systems, suggesting that hydrological regime acts as a directional rather than deterministic filter. RLQ analysis further revealed coherent multivariate covariation between environmental gradients and coordinated trait syndromes, whereas individual trait–environment associations identified by fourth-corner analysis were not significant after correction for multiple testing. Finally, spatial analyses showed that functional similarity declined more gradually with geographic distance than taxonomic similarity, indicating substantial functional redundancy across the regional macrophyte assemblage. Together, these results show that coordinated functional trait syndromes structure macrophyte diversity across flowing and impounded freshwater systems while maintaining functional redundancy that may enhance ecosystem stability.

## 1 Introduction

Understanding how ecological communities assemble along environmental gradients remains a central goal of ecology, particularly in the context of how biodiversity contributes to ecosystem stability under environmental change (Leibold et al., 2004; Tilman et al., 2006). In freshwater ecosystems, community organization is closely linked to physical processes, such as hydrology, geomorphology, and hydrological connectivity (Riis and Biggs, 2003). Metacommunity theory provides a useful framework for disentangling the relative roles of environmental filtering, dispersal, and spatial processes in shaping community composition within heterogeneous landscapes, and for evaluating how these processes influence the resilience and persistence of communities across space (Leibold et al., 2004; Heino et al., 2015).

Within freshwater ecosystems, aquatic macrophytes represent an informative model group for studying community assembly in freshwater systems. As primary producers rooted in, or closely tied to aquatic habitats, macrophytes respond strongly to local environmental conditions while also being constrained by dispersal pathways and hydrological connectivity (Riis and Biggs, 2003; Thomaz et al., 2007; Chambers et al., 2008). Their wide range of growth forms, mechanical properties, life-history strategies, and reproductive modes reflects adaptations to gradients in water depth, flow velocity, nutrient availability, and disturbance, making macrophyte communities well suited for trait-based analyses (Willby et al., 2000; Baattrup-Pedersen et al., 2015; Dalla Vecchia et al., 2020). Such trait variation not only reflects mechanisms of assembly but may also underpin the capacity of macrophyte communities to maintain function under hydrological variability and disturbance.

Trait-based ecology has increasingly been adopted as a means of linking community composition to underlying ecological mechanisms (Lavorel and Garnier, 2002; McGill et al., 2006). By focusing on functional traits (i.e., morphological, physiological, or life-history characteristics that influence species performance), trait-based approaches aim to move beyond species identity toward more generalizable patterns of community assembly and ecosystem functioning (Lavorel and Garnier, 2002; Violle et al., 2007; Díaz et al., 2016). Functional diversity and the distribution of trait syndromes are increasingly viewed as key determinants of ecosystem stability, as overlapping yet distinct functional strategies may buffer communities against environmental fluctuations (Loreau et al., 2001; Elmqvist et al., 2003). In freshwater ecology, trait-based studies suggest that macrophyte assemblages are often structured by coordinated trait syndromes rather than by isolated traits, reflecting integrated responses to multiple environmental constraints (Willby et al., 2000; Alahuhta et al., 2019).

Recent conceptual developments further emphasize that ecological strategies are best understood through coordinated combinations of traits rather than isolated attributes, as such syndromes capture integrated responses of organisms to environmental gradients (Sánchez-Martínez et al., 2024; Gutiérrez-Cánovas et al., 2024). These perspectives have stimulated growing interest in multivariate trait-based approaches capable of identifying coordinated functional strategies within freshwater communities.

Among the key environmental drivers shaping macrophyte assemblages, hydrological regime is particularly important (Poff et al., 1997; Riis and Biggs, 2003; Bornette and Puijalon, 2011). Flow velocity, water-level stability, and disturbance frequency influence not only species occurrence but also the dominance of particular functional strategies, such as submerged versus emergent growth forms, flexible versus rigid morphologies, and sexual versus vegetative reproduction (Willby et al., 2000; Bornette and Puijalon, 2011). However, recent trait-based studies suggest that hydrological regime often functions as a directional, rather than deterministic filter, shifting the relative abundance of functional strategies while still allowing substantial overlap among communities (Alahuhta and Heino, 2013; García-Girón et al., 2020). This may be particularly evident at regional scales, where shared species pools and dispersal can reduce sharp functional separation between habitat types, thereby maintaining functional overlap that could enhance system-level stability across hydrological context (Heino et al., 2015).

Methodological work has emphasized that trait-environment relationships are often multivariate and that simple pairwise trait-environment tests may inflate type I error, leading to overinterpretation of individual traits (Dray et al., 2014; ter Braak et al., 2018). Multivariate frameworks such as RLQ and fourth-corner analyses integrate environmental variables, species composition, and functional traits in a unified analytical approach for examining trait-environment linkages (Dolédec et al., 1996; Legendre et al., 1997; Dray et al., 2014). These methods are particularly useful for detecting coordinated trait syndromes and for reducing the risk of overinterpreting single-trait effects, while allowing inference about how trait combinations respond collectively to environmental gradients (ter Braak et al., 2018).

At broader spatial extents, large-scale syntheses have shown that the relative importance of environmental filtering and spatial processes in macrophyte metacommunities varies substantially among regions, highlighting strong context dependency in community assembly (Heino et al., 2015; Alahuhta et al., 2018). Despite these advances, the joint consideration of functional trait structure, hydrological regime, and spatial turnover in macrophyte metacommunities remains comparatively uncommon within a single analytical framework, particularly across networks spanning both flowing and impounded waters. In regulated landscapes, rivers, artificial canals, lakes, and reservoirs often coexist within the same region, yet differ markedly in hydrology, geomorphology, and human influence. Understanding how macrophyte trait composition responds to such heterogeneity is important for advancing trait-based freshwater ecology and for assessing how functional diversity and redundancy may contribute to stability of increasingly modified aquatic systems.

To address this, we use a trait-based framework to examine macrophyte community assembly across flowing and impounded freshwater systems in Serbia. Specifically, we integrate analyses of functional trait space, multivariate trait–environment relationships, and spatial turnover in order to evaluate how hydrological regime and environmental gradients jointly structure macrophyte functional composition across the regional freshwater network. To achieve this, we: (i) characterize the functional trait space of the regional macrophyte species pool using multivariate ordination of species functional traits; (ii) assess whether community functional composition differs between flowing and impounded systems by projecting site-level communities into this trait space; (iii) examine multivariate relationships between traits and environmental gradients using integrated RLQ and fourth-corner approaches; and (iv) compare patterns of functional and taxonomic turnover over geographic distance to evaluate the extent of functional redundancy across the study region. Together, these analyses provide a mechanistic perspective on how hydrological regime, environmental gradients, and spatial processes interact to shape macrophyte trait composition in heterogeneous freshwater systems and how these patterns may influence functional redundancy and potential stability at regional scales.

## 2 Materials and methods

### 2.1 Study area and macrophyte data

Macrophyte surveys were conducted in 2017, 2019, and 2024 within the framework of Serbia’s National Monitoring Programme for ecological classification of surface waters, implemented in accordance with the European Water Framework Directive (European Commission, 2000). Sampling followed European standard EN 14184 for macrophyte assessment in running waters (CEN (European Committee for Standardization), 2014) and European standard EN 15460 for macrophyte assessment in lakes (CEN (European Committee for Standardisation), 2007).

**Figure 1:**
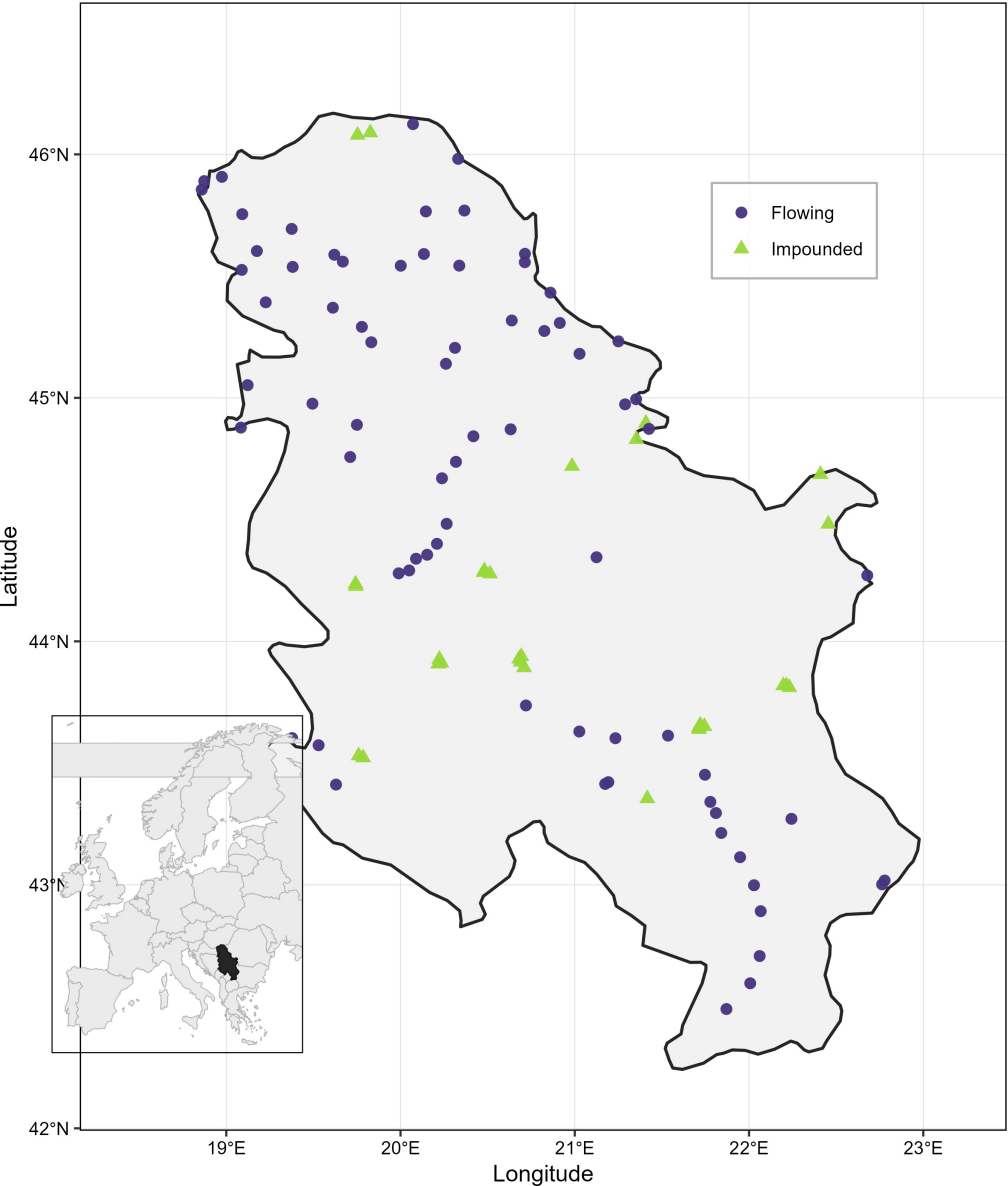
Location of the 102 sampling sites included in the study across Serbia. Symbols distinguish hydrological types, with flowing systems (rivers and artificial canals) shown as circles and impounded (lakes and reservoirs) as triangles.

In the monitoring programme, a sampling site represents a predefined monitoring section of a waterbody (river, canal, lake, or reservoir) defined for WFD ecological status assessment. Within each site, macrophytes were surveyed along longitudinal belt transects (survey units) that were 100, 500, or 1,000 m long depending on waterbody size and morphology. At each site, a minimum of three survey units were surveyed along each bank. Each site was surveyed once within the monitoring programme and therefore represents a single spatial observation rather than repeated temporal sampling. Multiple monitoring sites could occur within the same river or canal where separate WFD monitoring sections were defined. Across all surveys, 102 sites distributed across Serbia were included in the analyses (**Figure 1**).

Species abundance within each survey unit was estimated using the five-degree Kohler scale (Kohler, 1978). All aquatic growth forms were recorded, including submerged, floating-leaved, free-floating, and emergent species. Across all sites, a total of 81 macrophyte species were observed. For the purposes of the present analyses, species abundance values from all survey units within a site were averaged to obtain a single site-level abundance value, so that each site represents one macrophyte community observation in the site × species matrix used for subsequent analyses.

### 2.2 Environmental variables

Environmental variables were selected to represent major physicochemical, hydro-morphological, and landscape gradients commonly linked to macrophyte community structure at local and regional scales (Riis and Biggs, 2003; Chambers et al., 2008; Bornette and Puijalon, 2011; Alahuhta and Heino, 2013). Physicochemical variables included annual mean values of water temperature, dissolved oxygen, pH, electrical conductivity, dissolved inorganic nitrogen (DIN), and total phosphorus (TP). Environmental variables were obtained from the national surface water monitoring database maintained by the Serbian Environmental Protection Agency. Measurements were taken at monitoring stations corresponding to the macrophyte sampling sites or at the nearest official monitoring station within the same waterbody segment. These variables represent standardized monitoring measurements collected within the same monitoring programme and were matched to the macrophyte survey locations and to the year of biological sampling for each site. Hydro-morphological and landscape variables included channel width, bank slope, riparian zone width, elevation, surrounding land-cover richness, and average degree of hemeroby as an index of human disturbance. Land cover information was derived from CORINE Land Cover data (https://land.copernicus.eu/en/products/corine-land-cover) and used to calculate land-cover richness and average hemeroby values (Waltz and Stein, 2014). Channel width and riparian zone width were estimated using high-resolution satellite imagery. Summary statistics of environmental variables by hydrological type (**Supplementary Table 1**), and Spearman correlation matrix of environmental variables used in trait-environment analyses (**Supplementary Figure 1**) are provided in the **Supplementary Material**. Prior to analyses, environmental variables were inspected for missing values and extreme outliers. Numeric variables were standardized to zero-mean and unit variance for multivariate analyses.

### 2.3 Functional trait data

Functional traits were compiled for all macrophyte species recorded in the surveys. The trait framework encompassed growth form, mechanical and morphological characteristics, life-history strategy, and modes of reproduction (**Table 1**). Trait information was obtained from published literature and trait databases, including LEDA (Kleyer et al., 2008) and TRY (Kattge et al., 2020) where available, and supplemented by regional expert knowledge where necessary. Traits were coded as binary or ordinal variables, with ordinal traits representing increasing expression or intensity. Multiple trait states were allowed where ecologically appropriate (e.g. heterophyllous species or multiple reproductive modes). An explicit quality control workflow was applied to the species × traits matrix (**Supplementary Table 2**), including checks for missing values, allowed value ranges, low trait variation, and logical consistency among traits. Traits with zero variance across the species pool were excluded prior to analysis.

**Table 1:** Functional traits used to characterize aquatic macrophyte species.

Trait group	Trait	Code	Type	Description
Growth form	Submerged	sbm	Binary	Fully submerged growth form
	Floating	flt	Binary	Free-floating of floating-leaved growth form
	Emergent	emg	Binary	Emergent growth form
	Amphibious habit	amph	Binary	Grow in aquatic and terrestrial conditions
Life-history	Annual	annl	Binary	Annual life cycle
	Perennial	prnnl	Binary	Perennial life cycle
Mechanical/	Anchoring strength	anchr	Ordinal (0-2)	Degree of anchorage to substrate
morphological	Body flexibility	bdflx	Ordinal (1-3)	Flexibility of shoots and leaves
	Str. complexity	strcmplx	Ordinal (1-3)	Degree of plant body structural complexity
	Leaf texture	lftxt	Ordinal (1-3)	Soft to rigid leaf tissues
	Leaf area	lfarea	Ordinal (1-4)	Leaf size classes
	Nodal rooting	rtnd	Binary	Rooting at stem nodes
Persistence	Clonality	clnlty	Ordinal (0-2)	Capacity for vegetative clonal growth
Reproduction	Rhizomes	rhzm	Binary	Vegetative reproduction via rhizomes
	Seeds	sds	Binary	Sexual reproduction by seeds
	Fragmentation	frgm	Binary	Vegetative reproduction via fragmentation
	Budding	bdng	Binary	Vegetative reproduction via buds
	Turions	trns	Binary	Production of turions
	Stolons	stln	Binary	Vegetative reproduction via stolons
	Tubers	tbrs	Binary	Vegetative reproduction via tubers
	Spores	sprs	Binary	Reproduction via spores

Traits describe growth form, mechanical properties, life-history strategy and modes of reproduction. Binary traits are coded as 0/1, ordinal traits increase with trait intensity, and multiple states are allowed where ecologically appropriate.

### 2.4 Species functional trait space

Functional relationships among species were quantified using Gower distances, which allow inclusion of mixed data types (Gower, 1971). A Principal Coordinates Analysis (PCoA) was performed on the resulting distance matrix to describe the multivariate structure of the species functional trait space. Rather than calculating univariate functional diversity indices (e.g., functional richness or Rao’s quadratic entropy), we focused on the explicit structure of multivariate trait space and the projection of communities within that space to identify coordinated trait syndromes and their environmental filtering. To aid interpretation, trait vectors were fitted onto the PCoA ordination using the *envfit* procedure, providing correlations between individual traits and ordination axes. These vectors were used for descriptive purposes only.

### 2.5 Community-level functional composition

Community functional composition was characterized by projecting site-level communities into the species functional trait space using community-weighted positions, calculated as abundance-weighted mean species scores along the first two PCoA axes. Kohler abundance values were treated as relative weights. Differences in community functional composition between hydrological types were tested using PERMANOVA based on Euclidean distances between site positions in trait space (Anderson, 2001), with significance assessed using 9999 permutations.

### 2.6 Trait-environment relationships

Trait-environment relationships were analyzed using a combination of RLQ analysis and fourth-corner tests, which jointly evaluate environmental variables (R), species composition (L), and functional traits (Q) (Dolédec et al., 1996; Legendre et al., 1997). RLQ analysis was used to describe the dominant structure of joint trait-environment covariation across sites, and the resulting ordination was visualized to illustrate the main gradients linking environmental conditions, species composition, and functional traits. RLQ analysis therefore summarizes multivariate relationships between environmental gradients and coordinated trait combinations rather than testing individual trait-environment associations. Species abundance data were Hellinger-transformed prior to analysis to reduce the influence of dominant species and to meet the assumptions of ordination methods (Legendre and Gallagher, 2001). Row and column weights derived from correspondence analysis of the species composition matrix were applied consistently across R, L, and Q tables to ensure compatibility among ordinations. Fourth-corner analyses were conducted using permutation model 6, with 9999 permutations, which accounts for the dependence structure among the three tables by permuting both sites and species (Dray et al., 2014). False discovery rate correction was applied to control for multiple testing.

### 2.7 Spatial turnover of taxonomic and functional composition

Geographic coordinates of all sampling sites were recorded in the field using handheld GPS units. Pairwise geographic distances between sites were calculated as great-circle distances based on these coordinates.

Spatial turnover in community composition was examined using distance-decay analyses by relating pairwise taxonomic and functional similarity to geographic distance (Soininen et al., 2007). Taxonomic similarity was calculated as one minus Bray-Curtis dissimilarity based on Hellinger-transformed species abundance data. Functional similarity was derived from Euclidean distances between site scores in functional trait space.

Spatial information was used exclusively to quantify geographic distance among sites and was not included as an explanatory predictor in the multivariate analyses, as the primary objective was to examine patterns of functional trait structure and trait-environment relationships.

All analyses were conducted in R (version 4.5.2; R Core Team, 2025). Multivariate and trait-based analyses were performed primarily using packages *vegan* (Oksanen et al., 2025) and *ade4* (Dray and Dufour, 2007). R script used for analyses is provided in the **Supplementary Material**.

## 3 Results

Across 102 surveyed sites, macrophyte communities comprised 81 species from the regional species pool. Species richness per site ranged from 1 to 24 species, with a mean of 8.6 ± 5.0 species. Communities in flowing systems contained 8.9 ± 4.6 species per site (range 1-20), while impounded systems contained 8.0 ± 5.8 species per site (range 1-24). Most sites were therefore characterized by relatively species-poor assemblages dominated by a limited number of macrophyte taxa, although some sites supported more diverse communities.

Environmental conditions varied across sites but generally fell within the typical range reported for temperate freshwater systems. Water temperature ranged from 7.9 to 20.7 °C, conductivity from 126 to 1529 µS cm^-1^, dissolved oxygen from 5.2 to 18.4 mg L^-1^, total phosphorus from 0.018 to 0.75 mg L^-1^, and dissolved inorganic nitrogen from 0.28 to 6.19 mg L^-1^, reflecting moderate environmental heterogeneity across the surveyed habitats (**Supplementary Table 1**). This descriptive overview provides context for the trait-based analyses of functional structure presented below.

### 3.1 Functional structure of the macrophyte trait space

**Figure 2:**
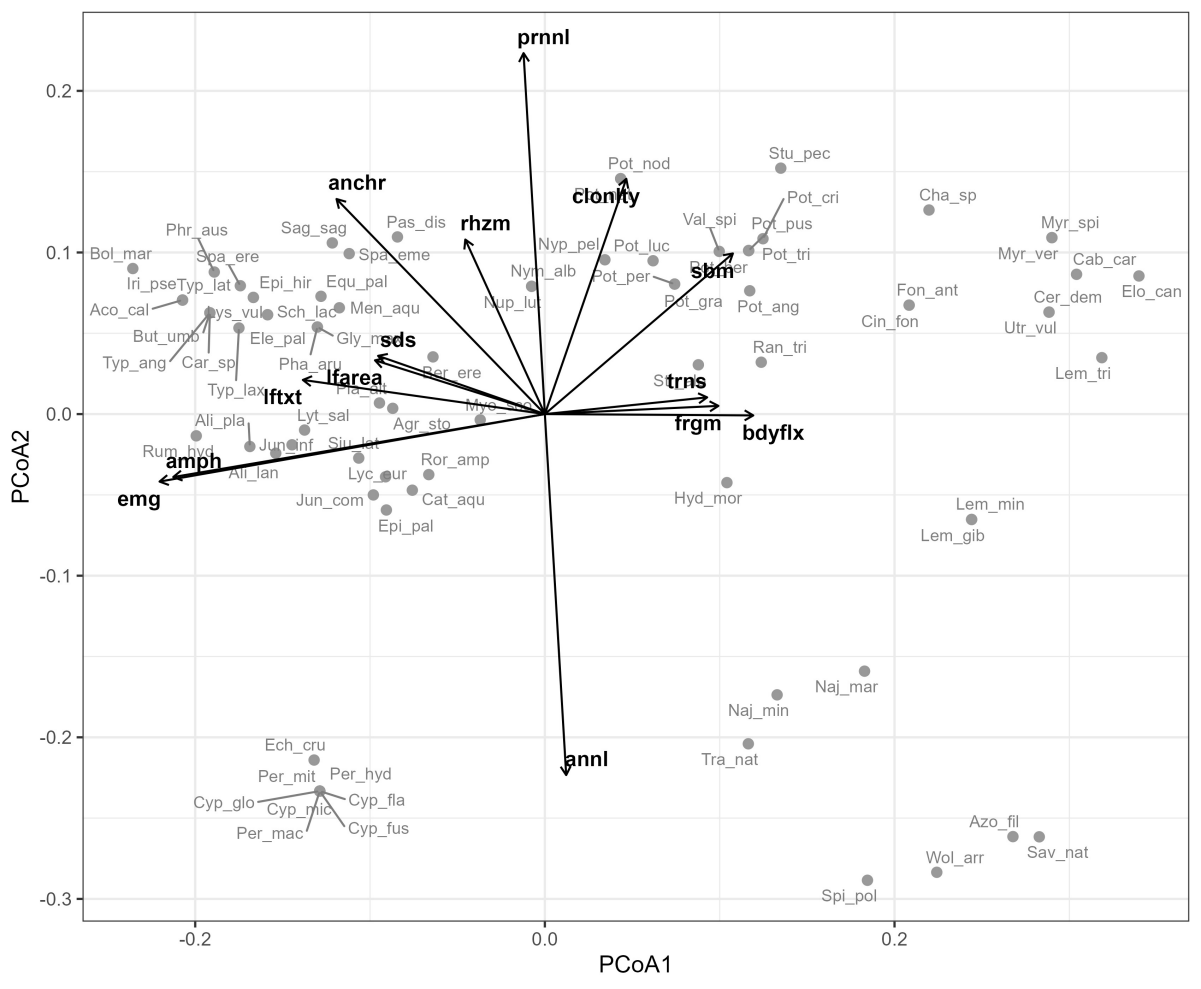
Principal coordinates analysis (PCoA) of aquatic macrophyte species based on Gower distances calculated from the species × traits matrix. Points represent species and are labeled by species codes. Black arrows show fitted trait vectors (*envfit*), indicating the direction and strength of correlations between traits and ordination axes; only traits with *r^2^* > 0.3 are displayed. The first axis represents a gradient from emergent and amphibious, rigid, seed-reproducing strategies to submerged, flexible species relying on vegetative propagation, while the second axis reflects a life-history persistence gradient contrasting annual and perennial, clonal strategies.

The Principal Coordinates Analysis (PCoA) of species functional traits based on Gower distances revealed a clear and ecologically interpretable structure of the regional macrophyte trait space (**Figure 2**). The first two axes accounted for approximately 45% of total functional variation (PCoA1 ≈ 29%, PCoA2 ≈ 16%) and were therefore retained for further interpretation.

PCoA1 represented a dominant gradient in growth form and mechanical strategy. Negative scores were associated with emergent and amphibious species characterized by rigid tissues, larger leaves and predominantly sexual reproduction by seeds, whereas positive scores corresponded to fully submerged species with high body flexibility and reliance on vegetative propagules such as fragmentation and turions (**Figure 2**; **Supplementary Table 3**).

PCoA2 captured a life-history persistence gradient. Positive scores were associated with perennial, strongly clonal and well anchored species, often possessing rhizomatous growth, while negative scores were dominated by annual strategies. Annual and perennial life cycles showed nearly complete opposition along this axis, indicating a clear separation between short-lived and persistent functional strategies (**Figure 2**). The two axes were largely orthogonal, suggesting that growth form and mechanical traits were decoupled from life-history persistence across the species pool.

### 3.2 Functional differentiation of communities between hydrological types

**Figure 3:**
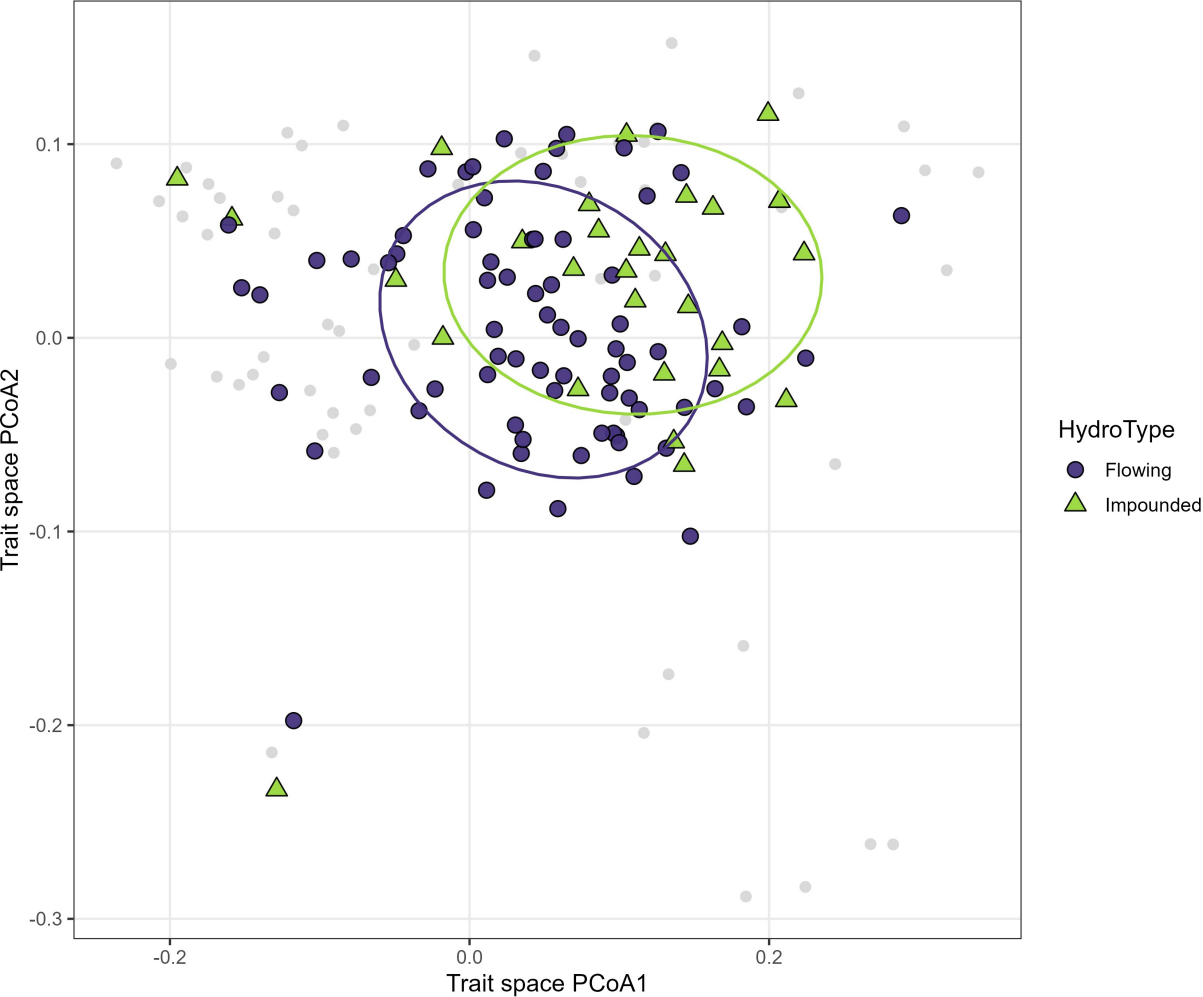
Projection of site-level macrophyte communities into the species functional trait space using community-weighted positions. Gray points indicate species scores from the trait PCoA, whereas symbols represent site scores and are distinguished by hydrological type (Flowing vs Impounded). Ellipses denote 68% confidence regions for each hydrological type.

Projection of site-level macrophyte communities into the species trait space using community-weighted positions revealed a significant, but partial differentiation between flowing and impounded systems (**Figure 3**). Most sites from both hydrological types occupied the upper portion of the trait space (PCoA2 > -0.1), indicating a general dominance of perennial and clonal strategies across the study region.

Despite substantial overlap between groups, impounded sites tended to be shifted toward the positive ends of both PCoA axes, corresponding to communities dominated by submerged, flexible species with strong vegetative persistence. In contrast, flowing sites were more centrally distributed along the mechanical strategy gradient, reflecting greater functional heterogeneity in growth form and reproductive models (**Supplementary Figure 2**). Only a small number of sites from either hydrological type occupied lower left portion of the trait space, associated with annual and emergent strategies.

Hydrological type explained a small but significant proportion of variation in community functional composition (PERMANOVA: R^2^ = 0.038, F = 3.90, p = 0.029; **Supplementary Table 4**), indicating a consistent directional influence of hydrological regime on trait composition despite extensive overlap among communities.

### 3.3 Multivariate trait-environment relationships

**Figure 4:**
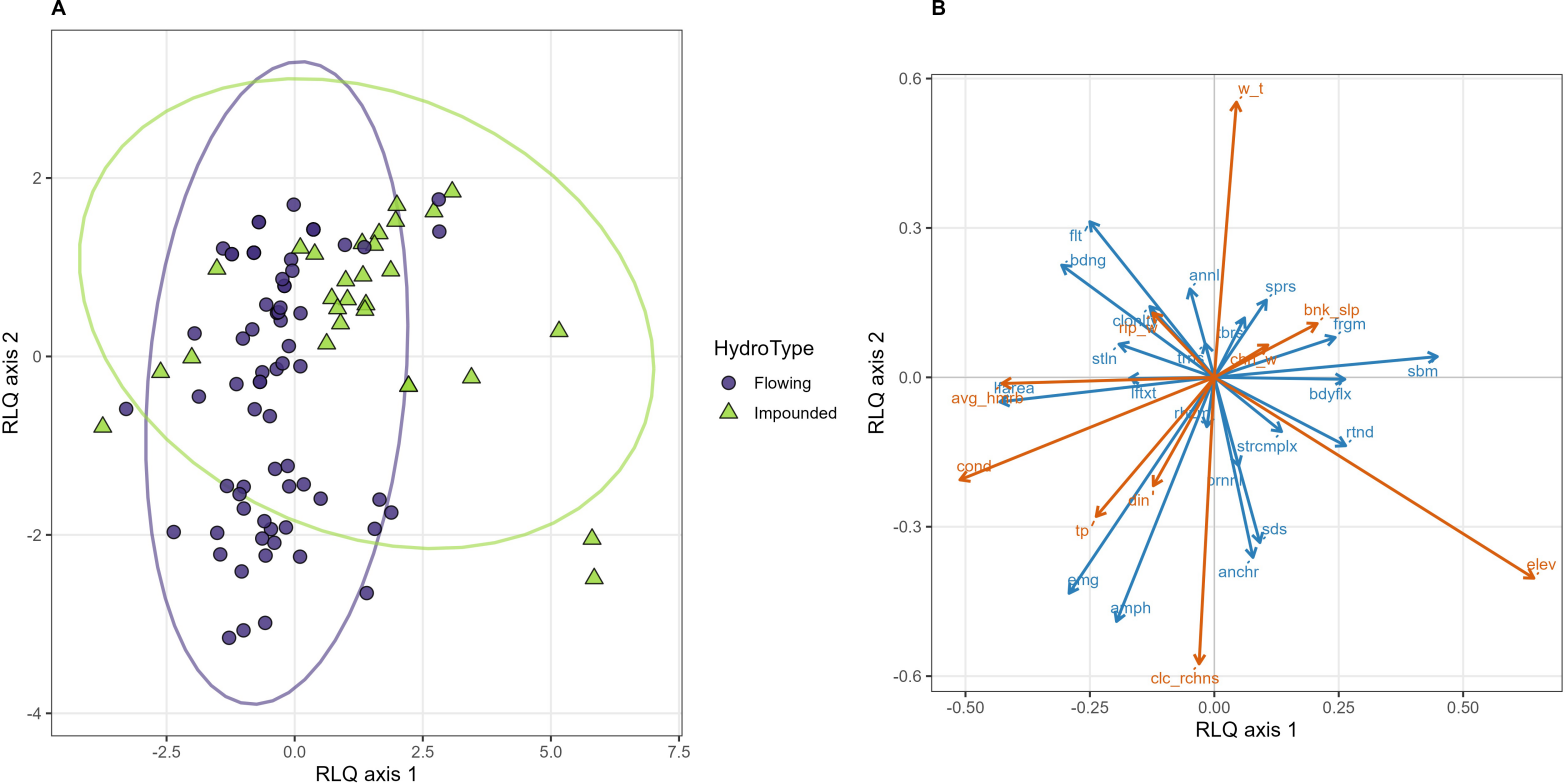
RLQ ordination illustrating joint relationships among environmental variables, macrophyte community composition, and functional traits. **(A)** Site scores along the first two RLQ axes, with symbols indicating hydrological type. **(B)** Trait–environment biplot showing the environmental gradients and functional traits associated with the first two RLQ axes.

RLQ analysis revealed a clear structure of joint covariation between environmental gradients, species composition, and functional traits. The first two RLQ axes captured most of the shared structure among the environmental (R), species (L), and trait (Q) tables (eigenvalues: RLQ axis 1 = 0.437; RLQ axis 2 = 0.287; **Supplementary Table 5**). Because RLQ analysis maximizes the covariance between environmental variables and species traits mediated by community composition, the resulting axes represent coordinated trait–environment gradients rather than independent ordinations of traits or environmental variables. The ordination therefore summarizes how environmental gradients structure macrophyte functional strategies across sites (**Figure 4**; **Supplementary Figure 3**). The distribution of macrophyte species within this joint trait–environment space is shown in **Supplementary Figure 4**.

The first RLQ axis represented a dominant gradient linking physicochemical conditions and landscape context with macrophyte growth-form strategies. On the environmental side, negative scores were associated with higher conductivity, nutrient concentrations, and human disturbance (hemeroby), whereas positive scores corresponded primarily to higher-elevation sites. In the trait–environment biplot (**Figure 4B**), submerged growth form, body flexibility, and vegetative propagation traits aligned with environmental vectors associated with higher elevation and lower conductivity, whereas emergent and amphibious growth forms together with larger leaf area aligned with gradients of higher nutrient concentration and conductivity. This contrast indicates that environmental gradients primarily redistributed communities along a mechanical and growth-form strategy axis, separating submerged flexible species from emergent or structurally complex forms. This pattern broadly corresponds to the principal growth-form gradient identified in the species functional trait space (**Figure 2**).

The second RLQ axis primarily reflected variation in life-history and reproductive strategies. Positive scores were associated with floating and short-lived strategies, whereas negative scores corresponded to perennial, anchored, and amphibious species. The distribution of sampling sites along the first two RLQ axes (**Figure 4A**) therefore reflects the combined influence of environmental gradients on coordinated macrophyte functional strategies.

Fourth-corner analyses identified several ecologically plausible trait–environment associations when unadjusted significance levels were considered (**Supplementary Table 6**; **Supplementary Figure 5**). However, none of these associations remained significant after correction for multiple testing using false discovery rate adjustment, indicating that functional responses to environmental gradients were distributed across multiple traits rather than driven by isolated trait–environment links. Accordingly, fourth-corner results were treated as exploratory and interpreted in conjunction with the multivariate RLQ patterns.

### 3.4 Spatial turnover of taxonomic and functional composition

**Figure 5:**
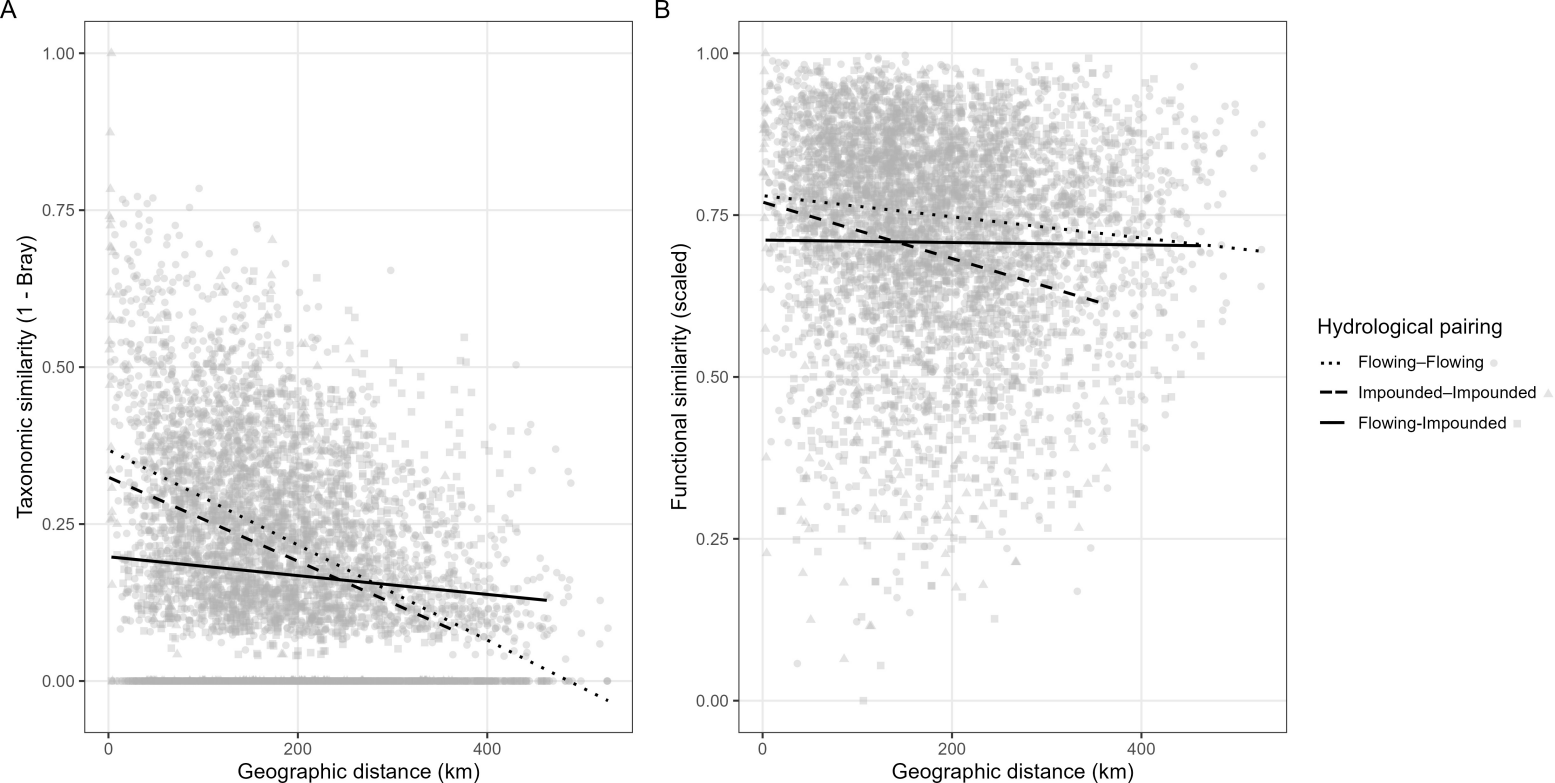
Distance-decay relationship of **(A)** taxonomic similarity and **(B)** functional similarity between pairs of sites across geographic distance. Taxonomic similarity was calculated as 1 – Bray-Curtis dissimilarity based on species composition, while functional dissimilarity was derived from Euclidean distances between site positions in trait space. Points represent site pairs and lines indicate fitted linear trends.

Comparisons of taxonomic and functional similarity across geographic distance revealed clear patterns of spatial turnover (**Figure 5**). Taxonomic similarity declined more rapidly with increasing geographic distance than functional similarity, indicating that species replacement across sites was not accompanied by proportional shifts in functional composition and suggesting partial functional redundancy among macrophyte communities across the study region.

Distance–decay relationships further differed between hydrological types (**Figure 5**). Functional similarity among impounded–impounded site pairs declined more rapidly with geographic distance than among flowing–flowing pairs, indicating stronger spatial differentiation of functional composition within impounded systems. In contrast, functional similarity in flowing systems was maintained over broader spatial extents, consistent with the greater longitudinal connectivity characteristic of river networks. This spatial turnover pattern complements the trait–site projection (**Figure 3**), where impounded sites occupy a relatively constrained region of functional space but diverge more strongly with increasing geographic separation, whereas flowing sites span a broader range of strategies yet exhibit more gradual spatial decay of functional similarity.

Taken together, these results indicate that macrophyte communities across flowing and impounded freshwater systems are structured by a limited number of dominant functional gradients while retaining substantial overlap in trait composition across space and hydrological contexts, suggesting that species turnover occurs largely within a shared set of functional strategies.

## 4 Discussion

### 4.1 Functional syndromes as the primary axis of macrophyte community structure

Our analyses indicate that macrophyte community structure across the study region is organized primarily by coordinated functional syndromes rather than by isolated trait responses. Both the species trait ordination and the RLQ analysis revealed two dominant and largely independent gradients structuring the functional space of the regional species pool. The first gradient contrasted emergent and amphibious species characterized by rigid tissues and seed-based reproduction with submerged, flexible species relying predominantly on vegetative propagation, while the second gradient separated annual from perennial persistence strategies. Together, these axes capture the principal dimensions through which macrophytes respond to environmental variation across flowing and impounded freshwater systems. The RLQ analysis further supports this interpretation by showing that these functional dimensions are not merely patterns within species trait space but are directly aligned with environmental gradients across sites. In particular, the dominant RLQ axis linked physicochemical conditions and landscape context with a shift between submerged flexible strategies and emergent or structurally complex growth forms, indicating that coordinated trait syndromes mediate the response of macrophyte communities to environmental heterogeneity.

At the community level, most sites were dominated by perennial and clonal strategies irrespective of hydrological type, indicating that persistence-oriented life-history strategies form a common baseline across the regional macrophyte assemblage. Such strategies are widely interpreted as adaptations to the recurrent disturbance regimes and environmental variability characteristic of freshwater habitats (Riis and Biggs, 2003; Bornette and Puijalon, 2011; Dalla Vecchia et al., 2020). Rather than reflecting local idiosyncrasies, this pattern likely arises from the combined effects of regional species pools, hydrological connectivity, and repeated exposure of aquatic habitats to both natural and anthropogenic disturbances. Within this baseline, functional differentiation among communities was expressed mainly along the mechanical and growth-form gradient. This suggests that variation in flow conditions, water depth, and habitat structure primarily determines which functional strategies are locally favored, while life-history persistence remains relatively conserved across systems. Such patterns align with trait-based frameworks that emphasize coordinated trait combinations as the primary currency of community assembly (Willby et al., 2000; Lavorel and Garnier, 2002; Dalla Vecchia et al., 2020; Gutiérrez-Cánovas et al., 2024).

Beyond confirming the importance of coordinated trait strategies, this study extends previous trait-based macrophyte research by integrating functional trait structure, hydrological regime, and spatial turnover within a single analytical framework across flowing and impounded freshwater systems. Many earlier studies have examined these components separately, focusing either on trait–environment relationships or on spatial patterns of community composition (e.g., Alahuhta et al., 2018; Dalla Vecchia et al., 2020; García-Girón et al., 2020; Li et al., 2024; Alahuhta et al., 2025). By jointly analyzing trait syndromes, environmental gradients, and geographic distance across a regional monitoring network, our results provide a more integrated view of how functional strategies are filtered and redistributed across heterogeneous freshwater landscapes.

### 4.2 Hydrological regime as a directional filter

Hydrological regime exerted a statistically significant but modest influence on community functional composition. Flowing and impounded systems did not form discrete functional assemblages; instead, they showed a directional shift in trait space accompanied by substantial overlap. Communities in impounded systems tended to be associated with submerged, flexible, and vegetatively persistent strategies, whereas flowing systems encompassed a broader range of mechanical and reproductive strategies.

This pattern reinforces the view that hydrological regime can function as a directional filter rather than a deterministic one, particularly at regional scales where dispersal is high and species pools are shared (Poff et al., 1997; Riis and Biggs, 2003; Alahuhta and Heino, 2013; Bolpagni et al., 2024). Trait plasticity and broad ecological tolerances may allow many macrophyte species to persist across contrasting hydrological contexts, resulting in functional overlap despite consistent shifts in dominance (Bornette and Puijalon, 2011). Recent large-scale analyses of aquatic plant diversity similarly indicate that environmental gradients often shape macrophyte communities through broad effects on growth forms and ecological strategies rather than through strict habitat specialization (Alahuhta et al., 2018; Zhou et al., 2023).

Importantly, hydrological regime in this study was closely intertwined with geomorphological context, as flowing sites were mainly located in lowland rivers and canals, while impounded sites were often reservoirs embedded in upland valleys. Similar interactions between hydrology and physical settings have been noted in regional scale macrophyte studies, which caution against attributing community patterns to hydrology alone without considering geomorphology and landscape context (Chambers et al., 2008; Alahuhta and Heino, 2013). In this sense, the observed functional shifts likely reflect combined effects of flow regime, habitat structure, and environmental gradients associated with impoundment.

### 4.3 Distributed trait-environment relationships

Multivariate analyses revealed coherent structure in trait-environment covariation, as captured by RLQ ordination, linking hydro-morphological and physicochemical gradients with functional strategies (Dolédec et al., 1996; Dray et al., 2014). In contrast, individual trait-environment associations identified by fourth-corner analysis did not remain significant after correction for multiple testing.

This outcome reflects a well-recognized feature of trait–environment analyses in systems characterized by strong trait covariation and moderate environmental gradients, where multivariate ordination methods may reveal coordinated patterns even when individual trait–environment relationships do not remain significant after correction for multiple testing (Dray et al., 2014; ter Braak et al., 2018; Dalla Vecchia et al., 2020). In such settings, controlling for multiple comparisons may substantially reduce statistical power to detect individual trait-environment links, even when overall multivariate structure is clear (ter Braak et al., 2018). Accordingly, the clear structure observed in the RLQ ordination indicates that environmental gradients are associated with coordinated shifts in macrophyte functional strategies rather than with strong responses of individual traits. Recent syntheses in freshwater trait ecology similarly emphasize that trait–environment relationships are frequently expressed through coordinated trait combinations and context-dependent responses rather than through strong single-trait associations, highlighting the importance of multivariate approaches when interpreting functional patterns in aquatic communities (Gutiérrez-Cánovas et al., 2024). Rather than undermining trait-based inference, our results underscore the value of integrated frameworks that focus on multivariate trait syndromes and trait composition, rather than isolated trait effects (Legendre et al., 1997; Lavorel and Garnier, 2002; Dray et al., 2014; Sánchez-Martínez et al., 2024).

### 4.4 Functional redundancy and spatial turnover

Functional similarity declined more gradually with geographic distance than taxonomic similarity, indicating substantial functional redundancy across the regional macrophyte assemblage. Such redundancy has been proposed as a mechanism contributing to ecological stability, as overlapping functional strategies may buffer ecosystem processes against species turnover and environmental variability (Loreau et al., 2001; Elmqvist et al., 2003). From a metacommunity perspective, this pattern suggests that dispersal and regional species pools may allow different species to occupy similar functional niches across sites, thereby maintaining functional composition despite taxonomic turnover (Heino et al., 2015). Species replacement across space therefore did not translate directly into functional turnover, a pattern widely reported in freshwater metacommunity studies (Soininen et al., 2007; Heino et al., 2015; García-Girón et al., 2020; Zhou et al., 2023).

Distance-decay analyses further indicated that spatial patterns of functional turnover differed between hydrological types. Functional similarity among impounded sites declined more rapidly with geographic distance than among flowing sites, suggesting stronger spatial differentiation of trait composition within impounded systems. In contrast, functional similarity in flowing systems was maintained over broader spatial extents, indicating more gradual turnover along river networks. Cross-type comparisons showed relatively stable functional similarity across distance, consistent with baseline functional differentiation between flowing and impounded contexts. These patterns refine the interpretation derived from the distribution of communities in functional trait space. Although impounded sites occupied a comparatively constrained region of trait space overall, their functional composition diverged more strongly with increasing geographic separation. Flowing sites, by contrast, encompassed a wider range of strategies in trait space, yet exhibited more gradual spatial decay of functional similarity.

Taken together, these results suggest that hydrological regime influences not only the direction of functional filtering but also the spatial scaling of functional turnover. Impounded systems may be characterized by relatively consistent local filters combined with regional differentiation among reservoirs, whereas flowing systems integrate greater local heterogeneity within a spatially connected network that moderates functional divergence across distance (Thomaz et al., 2007; Heino et al., 2015). Similar contrasts have been observed in regulated river networks, where flow alteration and impoundment modify both taxonomic and functional turnover (Poff and Zimmerman, 2010).

### 4.5 Implications for trait-based freshwater ecology

Our results show that macrophyte community assembly across flowing and impounded freshwater systems is governed by a limited set of dominant functional gradients interacting with hydrological regime, geomorphology, and spatial context. Hydrological type exerted a consistent but non-exclusive influence on trait composition, producing directional shifts rather than discrete functional assemblages.

By combining an explicitly audited trait matrix with multivariate community and environmental analyses, this study reinforces the value of trait-based frameworks that emphasize coordinated strategies over single-trait explanations (Lavorel and Garnier, 2002; Alahuhta et al., 2019; Dalla Vecchia et al., 2020; Gutiérrez-Cánovas et al., 2024). Such approaches provide a useful basis for understanding macrophyte metacommunity structure in increasingly modified freshwater landscapes and for supporting management and conservation in systems facing ongoing environmental change, particularly where maintaining functional diversity and redundancy may enhance the capacity of communities to persist under hydrological alteration and disturbance.

Trait–environment relationships in freshwater macrophyte communities appear to operate primarily through coordinated trait syndromes shaped by hydrology, geomorphology, and spatial context, rather than through strong isolated trait responses. Our results therefore highlight the value of multivariate trait-based approaches for interpreting macrophyte metacommunity organization in regulated freshwater landscapes, where functional composition may remain partially buffered (i.e., functionally redundant) even as species turnover proceeds across space and habitat types. Despite growing interest in functional ecology, aquatic plants remain underrepresented in global trait databases and syntheses (Bolpagni et al., 2024). Expanding trait datasets and trait-based analyses across spatial scales will be essential for advancing freshwater plant macroecology and improving our ability to predict community responses to environmental change.

## Data Availability

The raw data supporting the conclusions of this article will be made available by the authors, without undue reservation.
