# Supplementary material for: Functional trait syndromes structure macrophyte diversity and functional redundancy across flowing and impounded freshwater systems
Source: Front Plant Sci. 2026 Apr 21;17:1815637. doi: 10.3389/fpls.2026.1815637 (PMC13139183; doi:10.3389/fpls.2026.1815637)
Supplement: Supplementary file 1 [file DataSheet1.docx]

Supplementary Material

# Supplementary Tables and Figures

## Supplementary Tables

**Table S1** Summary statistics (mean, standard deviation, median) of environmental variables for Flowing and Impounded sites.

| Environmental variable | Code |  | Flowing | Impounded |
| --- | --- | --- | --- | --- |
|  |  | n | 73 | 29 |
| Dissolved inorganic nitrogen | DIN | mean | 1.21 | 0.88 |
|  |  | sd | 0.49 | 1.09 |
|  |  | med | 1.01 | 0.58 |
| Total phosphorus | TP | mean | 0.21 | 0.14 |
|  |  | sd | 0.15 | 0.14 |
|  |  | med | 0.14 | 0.11 |
| Electrical conductivity | cond | mean | 463.83 | 404.31 |
|  |  | sd | 164.70 | 291.65 |
|  |  | med | 433.08 | 372.18 |
| Water temperature | w_t | mean | 14.05 | 16.24 |
|  |  | sd | 1.62 | 3.30 |
|  |  | med | 13.87 | 16.97 |
| Elevation | elev | mean | 140.44 | 275.96 |
|  |  | sd | 106.75 | 199.23 |
|  |  | med | 80.00 | 267.00 |
| Channel width | chn_w | mean | 114.97 | 458.46 |
|  |  | sd | 174.10 | 404.37 |
|  |  | med | 38.53 | 285.67 |
| Bank slope | bnk_slp | mean | 1.73 | 2.21 |
|  |  | sd | 0.52 | 0.66 |
|  |  | med | 2.00 | 2.50 |
| Riparian zone width | rip_w | mean | 105.78 | 2.25 |
|  |  | sd | 217.40 | 6.52 |
|  |  | med | 18.33 | 0.00 |
| Average degree of hemeroby | avg_hmrb | mean | 4.22 | 3.60 |
|  |  | sd | 0.79 | 1.12 |
|  |  | med | 4.40 | 3.50 |
| Corine Land Cover class richness | CLC_rchns | mean | 4.12 | 3.10 |
|  |  | sd | 2.40 | 1.54 |
|  |  | med | 4.00 | 3.00 |

**Table S2** Species × traits matrix used in all trait-based analyses. Rows represent macrophyte species and columns correspond to functional traits defined in Table 1.

| Code | Name | sbm | flt | emg | amph |
| --- | --- | --- | --- | --- | --- |
| Aco_cal | *Acorus calamus* L. | 0 | 0 | 1 | 1 |
| Agr_sto | *Agrostis stolonifera* L. | 0 | 0 | 1 | 1 |
| Ali_lan | *Alisma lanceolatum* With. | 0 | 0 | 1 | 1 |
| Ali_pla | *Alisma plantago-aquatica* L. | 0 | 0 | 1 | 1 |
| Azo_fil | *Azolla filiculoides* Lam. | 0 | 1 | 0 | 0 |
| Ber_ere | *Berula erecta* (Huds.) Coville | 1 | 0 | 1 | 1 |
| Bol_mar | *Bolboschoenus maritimus* (L.) Palla | 0 | 0 | 1 | 1 |
| But_umb | *Butomus umbellatus* L. | 0 | 0 | 1 | 1 |
| Cab_car | *Cabomba caroliniana* A. Gray | 1 | 0 | 0 | 0 |
| Car_sp | *Carex* sp. | 0 | 0 | 1 | 1 |
| Cat_aqu | *Catabrosa aquatica* (L.) Beauv. | 0 | 0 | 1 | 1 |
| Cer_dem | *Ceratophyllum demersum* L. | 1 | 0 | 0 | 0 |
| Cha_sp | *Chara* sp. | 1 | 0 | 0 | 0 |
| Cin_fon | *Cinclidotus fontinaloides* (Hedw.) P.Beauv. | 1 | 0 | 0 | 0 |
| Cyp_fla | *Cyperus flavescens* L. | 0 | 0 | 1 | 1 |
| Cyp_fus | *Cyperus fuscus* L. | 0 | 0 | 1 | 1 |
| Cyp_glo | *Cyperus glomeratus* L. | 0 | 0 | 1 | 1 |
| Cyp_mic | *Cyperus michellianus* (L.) Delile | 0 | 0 | 1 | 1 |
| Ech_cru | *Echinochloa crus-galii* (L.) P. Beauv. | 0 | 0 | 1 | 1 |
| Ele_pal | *Eleocharis palustris* (L.) Roem. et Schult. | 0 | 0 | 1 | 1 |
| Elo_can | *Elodea canadensis* Michx. | 1 | 0 | 0 | 0 |
| Epi_hir | *Epilobium hirsutum* L. | 0 | 0 | 1 | 1 |
| Epi_pal | *Epilobium palustre* L. | 0 | 0 | 1 | 1 |
| Equ_pal | *Equisetum palustre* L. | 0 | 0 | 1 | 1 |
| Fon_ant | *Fontinalis antipyretica* Hedw. | 1 | 0 | 0 | 0 |
| Gly_max | *Glyceria maxima* (Hartm.) Holomb. | 0 | 0 | 1 | 1 |
| Hyd_mor | *Hydrocharis morsus-ranae* L. | 0 | 1 | 0 | 0 |
| Iri_pse | *Iris pseudacorus* L. | 0 | 0 | 1 | 1 |
| Jun_com | *Juncus compressus* Jacq. | 0 | 0 | 1 | 1 |
| Jun_inf | *Juncus inflexus* L. | 0 | 0 | 1 | 1 |
| Lem_gib | *Lemna gibba* L. | 0 | 1 | 0 | 0 |
| Lem_min | *Lemna minor* L. | 0 | 1 | 0 | 0 |
| Lem_tri | *Lemna trisulca* L. | 1 | 0 | 0 | 0 |
| Lyc_eur | *Lycopus europaeus* L. | 0 | 0 | 1 | 1 |
| Lys_vul | *Lysimachia vulgaris* L. | 0 | 0 | 1 | 1 |
| Lyt_sal | *Lythrum salicaria* L. | 0 | 0 | 1 | 1 |
| Men_aqu | *Mentha aquatica* L. | 0 | 0 | 1 | 1 |
| Myo_sco | *Myosotis scorpioides* L. | 0 | 0 | 1 | 1 |
| Myr_spi | *Myriophyllum spicatum* L. | 1 | 0 | 0 | 0 |
| Myr_ver | *Myriophyllum verticilatum* L. | 1 | 0 | 0 | 0 |
| Naj_mar | *Najas marina* L. | 1 | 0 | 0 | 0 |
| Naj_min | *Najas minor* L. | 1 | 0 | 0 | 0 |
| Nup_lut | *Nuphar lutea* (L.) Sibth. & Sm. | 0 | 1 | 0 | 0 |
| Nym_alb | *Nymphaea alba* L. | 0 | 1 | 0 | 0 |
| Nyp_pel | *Nymphoides peltata* (S.G.Gmel) Kuntze | 0 | 1 | 0 | 0 |
| Pas_dis | *Paspalum distichum* L. | 1 | 0 | 1 | 1 |
| Per_hyd | *Persicaria hydropiper* (L.) Delabre | 0 | 0 | 1 | 1 |
| Per_mac | *Persicaria maculosa* Gray | 0 | 0 | 1 | 1 |
| Per_mit | *Persicaria mitis* (Schrank) Assenov | 0 | 0 | 1 | 1 |
| Pha_aru | *Phalaris arundinacea* L. | 0 | 0 | 1 | 1 |
| Phr_aus | *Phragmites australis* (Cav.) Trin. ex Steud. | 0 | 0 | 1 | 1 |
| Pla_alt | *Plantago altissima* L. | 1 | 0 | 1 | 1 |
| Pot_ang | *Potamogeton x angustifolius* J.Presl | 1 | 0 | 0 | 0 |
| Pot_ber | *Potamogeton berchtoldii* Fieber | 1 | 0 | 0 | 0 |
| Pot_cri | *Potamogeton crispus* L. | 1 | 0 | 0 | 0 |
| Pot_gra | *Potamogeton gramineus* L. | 1 | 0 | 0 | 0 |
| Pot_luc | Potamogeton lucens *L.* | 1 | 0 | 0 | 0 |
| Pot_nat | *Potamogeton natans* L. | 1 | 1 | 0 | 0 |
| Pot_nod | *Potamogeton nodosus* L. | 1 | 1 | 0 | 0 |
| Pot_per | *Potamogeton perfoliatus* L. | 1 | 0 | 0 | 0 |
| Pot_tri | *Potamogeton trichoides Cham. et Schltdl.* | 1 | 0 | 0 | 0 |
| Pot_pus | *Potamogeton pusillus* L. | 1 | 0 | 0 | 0 |
| Ran_tri | *Ranunculus trichophylus* Chaix ex Vill. | 1 | 0 | 0 | 1 |
| Ror_amp | *Rorippa amphibia* (L.) Besser | 0 | 0 | 1 | 1 |
| Rum_hyd | *Rumex hydrolapathum* Huds. | 0 | 0 | 1 | 1 |
| Sag_sag | *Sagittaria sagittifolia* L. | 1 | 0 | 1 | 1 |
| Sav_nat | *Salvinia natans* (L.) All. | 0 | 1 | 0 | 0 |
| Sch_lac | *Schoenoplectus lacustris* (L.) Palla | 0 | 0 | 1 | 1 |
| Siu_lat | *Sium latifolium* L. | 0 | 0 | 1 | 1 |
| Spa_eme | *Sparganium emersum* Rehmann. | 1 | 0 | 1 | 1 |
| Spa_ere | *Sparganium erectum* L. | 0 | 0 | 1 | 1 |
| Spi_pol | *Spirodela polyrrhiza* (L.) Schleid. | 0 | 1 | 0 | 0 |
| Str_alo | *Stratiotes aloides* L. | 1 | 1 | 0 | 0 |
| Stu_pec | *Stuckenia pectinata* (L.) Börner | 1 | 0 | 0 | 0 |
| Tra_nat | *Trapa natans* L. | 1 | 1 | 0 | 0 |
| Typ_ang | *Typha angustifolia* L. | 0 | 0 | 1 | 1 |
| Typ_lat | *Typha latifolia L.* | 0 | 0 | 1 | 1 |
| Typ_lax | *Typha laxmanii* Lepech. | 0 | 0 | 1 | 1 |
| Utr_vul | *Utricularia vulgaris* L. | 1 | 0 | 0 | 0 |
| Val_spi | *Vallisneria spiralis* L. | 1 | 0 | 0 | 0 |
| Wol_arr | *Wolffia arrhiza* (L.) Horkel ex Wimm. | 0 | 1 | 0 | 0 |

| Code | anchr | clonlty | lfarea | bdyflx | strcmplx | lftxt | rtnd | annl |
| --- | --- | --- | --- | --- | --- | --- | --- | --- |
| Aco_cal | 2 | 2 | 4 | 1 | 1 | 3 | 0 | 0 |
| Agr_sto | 1 | 2 | 2 | 2 | 2 | 2 | 1 | 0 |
| Ali_lan | 2 | 1 | 3 | 1 | 1 | 2 | 0 | 0 |
| Ali_pla | 2 | 1 | 4 | 1 | 1 | 2 | 0 | 0 |
| Azo_fil | 0 | 2 | 1 | 2 | 2 | 2 | 0 | 1 |
| Ber_ere | 2 | 1 | 2 | 2 | 3 | 2 | 0 | 0 |
| Bol_mar | 2 | 2 | 3 | 1 | 1 | 3 | 0 | 0 |
| But_umb | 2 | 2 | 3 | 1 | 1 | 3 | 0 | 0 |
| Cab_car | 1 | 2 | 1 | 3 | 3 | 1 | 1 | 0 |
| Car_sp | 2 | 2 | 3 | 1 | 1 | 3 | 0 | 0 |
| Cat_aqu | 1 | 1 | 2 | 2 | 2 | 2 | 0 | 0 |
| Cer_dem | 0 | 2 | 1 | 3 | 3 | 1 | 0 | 0 |
| Cha_sp | 2 | 2 | 1 | 2 | 2 | 3 | 1 | 0 |
| Cin_fon | 2 | 2 | 1 | 2 | 2 | 2 | 0 | 0 |
| Cyp_fla | 1 | 0 | 2 | 2 | 1 | 2 | 0 | 1 |
| Cyp_fus | 1 | 0 | 2 | 2 | 1 | 2 | 0 | 1 |
| Cyp_glo | 1 | 0 | 2 | 2 | 1 | 2 | 0 | 1 |
| Cyp_mic | 1 | 0 | 2 | 2 | 1 | 2 | 0 | 1 |
| Ech_cru | 1 | 0 | 3 | 2 | 2 | 2 | 0 | 1 |
| Ele_pal | 2 | 2 | 1 | 1 | 1 | 3 | 0 | 0 |
| Elo_can | 1 | 2 | 1 | 3 | 2 | 1 | 1 | 0 |
| Epi_hir | 2 | 2 | 4 | 1 | 2 | 2 | 0 | 0 |
| Epi_pal | 1 | 1 | 2 | 2 | 1 | 2 | 0 | 0 |
| Equ_pal | 2 | 2 | 1 | 1 | 2 | 3 | 0 | 0 |
| Fon_ant | 2 | 2 | 1 | 2 | 2 | 2 | 0 | 0 |
| Gly_max | 2 | 2 | 3 | 2 | 2 | 2 | 0 | 0 |
| Hyd_mor | 0 | 2 | 3 | 2 | 2 | 2 | 0 | 0 |
| Iri_pse | 2 | 2 | 4 | 1 | 1 | 3 | 0 | 0 |
| Jun_com | 1 | 1 | 1 | 2 | 1 | 3 | 0 | 0 |
| Jun_inf | 2 | 1 | 1 | 1 | 1 | 3 | 0 | 0 |
| Lem_gib | 0 | 2 | 1 | 2 | 1 | 1 | 0 | 0 |
| Lem_min | 0 | 2 | 1 | 2 | 1 | 1 | 0 | 0 |
| Lem_tri | 0 | 2 | 1 | 3 | 2 | 1 | 0 | 0 |
| Lyc_eur | 1 | 1 | 3 | 2 | 2 | 2 | 0 | 0 |
| Lys_vul | 2 | 2 | 4 | 1 | 2 | 2 | 0 | 0 |
| Lyt_sal | 2 | 1 | 3 | 1 | 2 | 2 | 0 | 0 |
| Men_aqu | 1 | 2 | 3 | 2 | 2 | 2 | 1 | 0 |
| Myo_sco | 1 | 2 | 2 | 2 | 2 | 2 | 1 | 0 |
| Myr_spi | 1 | 2 | 1 | 3 | 3 | 1 | 1 | 0 |
| Myr_ver | 1 | 2 | 1 | 3 | 3 | 1 | 1 | 0 |
| Naj_mar | 1 | 1 | 1 | 2 | 2 | 2 | 0 | 1 |
| Naj_min | 1 | 0 | 1 | 2 | 2 | 2 | 0 | 1 |
| Nup_lut | 2 | 2 | 4 | 1 | 2 | 2 | 0 | 0 |
| Nym_alb | 2 | 2 | 4 | 1 | 2 | 2 | 0 | 0 |
| Nyp_pel | 2 | 2 | 3 | 2 | 2 | 2 | 1 | 0 |
| Pas_dis | 2 | 2 | 3 | 2 | 2 | 2 | 1 | 0 |
| Per_hyd | 1 | 0 | 2 | 2 | 1 | 2 | 0 | 1 |
| Per_mac | 1 | 0 | 2 | 2 | 1 | 2 | 0 | 1 |
| Per_mit | 1 | 0 | 2 | 2 | 1 | 2 | 0 | 1 |
| Pha_aru | 2 | 2 | 3 | 2 | 2 | 2 | 0 | 0 |
| Phr_aus | 2 | 2 | 4 | 1 | 2 | 3 | 0 | 0 |
| Pla_alt | 1 | 1 | 3 | 1 | 1 | 2 | 0 | 0 |
| Pot_ang | 1 | 2 | 2 | 2 | 2 | 2 | 0 | 0 |
| Pot_ber | 1 | 2 | 1 | 3 | 1 | 1 | 0 | 0 |
| Pot_cri | 1 | 2 | 1 | 2 | 2 | 2 | 0 | 0 |
| Pot_gra | 1 | 2 | 2 | 2 | 2 | 2 | 0 | 0 |
| Pot_luc | 1 | 2 | 3 | 2 | 2 | 2 | 0 | 0 |
| Pot_nat | 2 | 2 | 4 | 1 | 2 | 2 | 0 | 0 |
| Pot_nod | 2 | 2 | 4 | 1 | 2 | 2 | 0 | 0 |
| Pot_per | 1 | 2 | 2 | 2 | 2 | 2 | 0 | 0 |
| Pot_tri | 1 | 2 | 1 | 3 | 1 | 1 | 0 | 0 |
| Pot_pus | 1 | 2 | 1 | 3 | 1 | 1 | 0 | 0 |
| Ran_tri | 1 | 1 | 1 | 3 | 3 | 1 | 0 | 0 |
| Ror_amp | 1 | 1 | 3 | 2 | 2 | 2 | 0 | 0 |
| Rum_hyd | 2 | 0 | 4 | 1 | 2 | 3 | 0 | 0 |
| Sag_sag | 2 | 2 | 4 | 2 | 2 | 2 | 0 | 0 |
| Sav_nat | 0 | 2 | 2 | 2 | 2 | 1 | 0 | 1 |
| Sch_lac | 2 | 2 | 1 | 1 | 1 | 3 | 0 | 0 |
| Siu_lat | 1 | 1 | 4 | 2 | 3 | 2 | 0 | 0 |
| Spa_eme | 2 | 2 | 3 | 2 | 1 | 2 | 0 | 0 |
| Spa_ere | 2 | 2 | 3 | 1 | 2 | 3 | 0 | 0 |
| Spi_pol | 0 | 2 | 2 | 1 | 1 | 2 | 0 | 1 |
| Str_alo | 1 | 2 | 3 | 1 | 1 | 3 | 0 | 0 |
| Stu_pec | 1 | 2 | 1 | 3 | 2 | 1 | 0 | 0 |
| Tra_nat | 1 | 0 | 4 | 2 | 2 | 2 | 0 | 1 |
| Typ_ang | 2 | 2 | 3 | 1 | 1 | 3 | 0 | 0 |
| Typ_lat | 2 | 2 | 4 | 1 | 1 | 3 | 0 | 0 |
| Typ_lax | 2 | 2 | 2 | 1 | 1 | 3 | 0 | 0 |
| Utr_vul | 0 | 2 | 1 | 3 | 3 | 1 | 0 | 0 |
| Val_spi | 2 | 2 | 3 | 3 | 1 | 1 | 0 | 0 |
| Wol_arr | 0 | 2 | 1 | 2 | 1 | 1 | 0 | 1 |

| Code | prnnl | rhzm | sds | frgm | bdng | trns | stln | tbrs | sprs |
| --- | --- | --- | --- | --- | --- | --- | --- | --- | --- |
| Aco_cal | 1 | 1 | 1 | 0 | 0 | 0 | 0 | 0 | 0 |
| Agr_sto | 1 | 0 | 1 | 0 | 0 | 0 | 1 | 0 | 0 |
| Ali_lan | 1 | 0 | 1 | 0 | 0 | 0 | 0 | 0 | 0 |
| Ali_pla | 1 | 0 | 1 | 0 | 0 | 0 | 0 | 0 | 0 |
| Azo_fil | 0 | 0 | 0 | 1 | 0 | 0 | 0 | 0 | 1 |
| Ber_ere | 1 | 0 | 1 | 0 | 0 | 0 | 0 | 0 | 0 |
| Bol_mar | 1 | 1 | 1 | 0 | 0 | 0 | 0 | 1 | 0 |
| But_umb | 1 | 1 | 1 | 0 | 0 | 0 | 0 | 0 | 0 |
| Cab_car | 1 | 0 | 0 | 1 | 0 | 0 | 0 | 0 | 0 |
| Car_sp | 1 | 1 | 1 | 0 | 0 | 0 | 0 | 0 | 0 |
| Cat_aqu | 1 | 0 | 1 | 0 | 0 | 0 | 0 | 0 | 0 |
| Cer_dem | 1 | 0 | 1 | 1 | 0 | 1 | 0 | 0 | 0 |
| Cha_sp | 1 | 0 | 0 | 1 | 0 | 0 | 0 | 0 | 1 |
| Cin_fon | 1 | 0 | 0 | 1 | 0 | 0 | 0 | 0 | 1 |
| Cyp_fla | 0 | 0 | 1 | 0 | 0 | 0 | 0 | 0 | 0 |
| Cyp_fus | 0 | 0 | 1 | 0 | 0 | 0 | 0 | 0 | 0 |
| Cyp_glo | 0 | 0 | 1 | 0 | 0 | 0 | 0 | 0 | 0 |
| Cyp_mic | 0 | 0 | 1 | 0 | 0 | 0 | 0 | 0 | 0 |
| Ech_cru | 0 | 0 | 1 | 0 | 0 | 0 | 0 | 0 | 0 |
| Ele_pal | 1 | 1 | 1 | 0 | 0 | 0 | 0 | 0 | 0 |
| Elo_can | 1 | 0 | 0 | 1 | 0 | 1 | 0 | 0 | 0 |
| Epi_hir | 1 | 1 | 1 | 0 | 0 | 0 | 0 | 0 | 0 |
| Epi_pal | 1 | 0 | 1 | 0 | 0 | 0 | 0 | 0 | 0 |
| Equ_pal | 1 | 1 | 0 | 0 | 0 | 0 | 0 | 0 | 1 |
| Fon_ant | 1 | 0 | 0 | 1 | 0 | 0 | 0 | 0 | 1 |
| Gly_max | 1 | 1 | 1 | 0 | 0 | 0 | 0 | 0 | 0 |
| Hyd_mor | 1 | 0 | 1 | 0 | 1 | 0 | 1 | 0 | 0 |
| Iri_pse | 1 | 1 | 1 | 0 | 0 | 0 | 0 | 0 | 0 |
| Jun_com | 1 | 0 | 1 | 0 | 0 | 0 | 0 | 0 | 0 |
| Jun_inf | 1 | 0 | 1 | 0 | 0 | 0 | 0 | 0 | 0 |
| Lem_gib | 1 | 0 | 0 | 0 | 1 | 1 | 0 | 0 | 0 |
| Lem_min | 1 | 0 | 0 | 0 | 1 | 1 | 0 | 0 | 0 |
| Lem_tri | 1 | 0 | 0 | 0 | 1 | 1 | 0 | 0 | 0 |
| Lyc_eur | 1 | 0 | 1 | 0 | 0 | 0 | 0 | 0 | 0 |
| Lys_vul | 1 | 1 | 1 | 0 | 0 | 0 | 0 | 0 | 0 |
| Lyt_sal | 1 | 0 | 1 | 0 | 0 | 0 | 0 | 0 | 0 |
| Men_aqu | 1 | 1 | 1 | 0 | 0 | 0 | 0 | 0 | 0 |
| Myo_sco | 1 | 0 | 1 | 1 | 0 | 0 | 0 | 0 | 0 |
| Myr_spi | 1 | 0 | 1 | 1 | 0 | 1 | 0 | 0 | 0 |
| Myr_ver | 1 | 0 | 1 | 1 | 0 | 1 | 0 | 0 | 0 |
| Naj_mar | 0 | 0 | 1 | 1 | 0 | 0 | 0 | 0 | 0 |
| Naj_min | 0 | 0 | 1 | 0 | 0 | 0 | 0 | 0 | 0 |
| Nup_lut | 1 | 1 | 1 | 0 | 0 | 0 | 0 | 0 | 0 |
| Nym_alb | 1 | 1 | 1 | 0 | 0 | 0 | 0 | 0 | 0 |
| Nyp_pel | 1 | 1 | 1 | 0 | 0 | 0 | 0 | 0 | 0 |
| Pas_dis | 1 | 0 | 1 | 0 | 0 | 0 | 1 | 0 | 0 |
| Per_hyd | 0 | 0 | 1 | 0 | 0 | 0 | 0 | 0 | 0 |
| Per_mac | 0 | 0 | 1 | 0 | 0 | 0 | 0 | 0 | 0 |
| Per_mit | 0 | 0 | 1 | 0 | 0 | 0 | 0 | 0 | 0 |
| Pha_aru | 1 | 1 | 1 | 0 | 0 | 0 | 0 | 0 | 0 |
| Phr_aus | 1 | 1 | 1 | 0 | 0 | 0 | 0 | 0 | 0 |
| Pla_alt | 1 | 0 | 1 | 0 | 0 | 0 | 0 | 0 | 0 |
| Pot_ang | 1 | 1 | 0 | 0 | 0 | 0 | 0 | 0 | 0 |
| Pot_ber | 1 | 1 | 1 | 0 | 0 | 0 | 0 | 0 | 0 |
| Pot_cri | 1 | 1 | 1 | 0 | 0 | 1 | 0 | 0 | 0 |
| Pot_gra | 1 | 1 | 1 | 0 | 0 | 0 | 0 | 0 | 0 |
| Pot_luc | 1 | 1 | 1 | 0 | 0 | 0 | 0 | 0 | 0 |
| Pot_nat | 1 | 1 | 1 | 0 | 0 | 0 | 0 | 0 | 0 |
| Pot_nod | 1 | 1 | 1 | 0 | 0 | 0 | 0 | 0 | 0 |
| Pot_per | 1 | 1 | 1 | 0 | 0 | 0 | 0 | 0 | 0 |
| Pot_tri | 1 | 1 | 1 | 0 | 0 | 0 | 0 | 0 | 0 |
| Pot_pus | 1 | 1 | 1 | 0 | 0 | 0 | 0 | 0 | 0 |
| Ran_tri | 1 | 0 | 1 | 1 | 0 | 0 | 0 | 0 | 0 |
| Ror_amp | 1 | 0 | 1 | 1 | 0 | 0 | 0 | 0 | 0 |
| Rum_hyd | 1 | 0 | 1 | 0 | 0 | 0 | 0 | 0 | 0 |
| Sag_sag | 1 | 0 | 1 | 0 | 0 | 0 | 1 | 1 | 0 |
| Sav_nat | 0 | 0 | 0 | 1 | 0 | 0 | 0 | 0 | 1 |
| Sch_lac | 1 | 1 | 1 | 0 | 0 | 0 | 0 | 0 | 0 |
| Siu_lat | 1 | 0 | 1 | 0 | 0 | 0 | 0 | 0 | 0 |
| Spa_eme | 1 | 1 | 1 | 0 | 0 | 0 | 0 | 0 | 0 |
| Spa_ere | 1 | 1 | 1 | 0 | 0 | 0 | 0 | 0 | 0 |
| Spi_pol | 0 | 0 | 0 | 0 | 1 | 1 | 0 | 0 | 0 |
| Str_alo | 1 | 0 | 1 | 0 | 1 | 0 | 0 | 0 | 0 |
| Stu_pec | 1 | 1 | 1 | 0 | 0 | 0 | 0 | 1 | 0 |
| Tra_nat | 0 | 0 | 1 | 0 | 0 | 0 | 0 | 0 | 0 |
| Typ_ang | 1 | 1 | 1 | 0 | 0 | 0 | 0 | 0 | 0 |
| Typ_lat | 1 | 1 | 1 | 0 | 0 | 0 | 0 | 0 | 0 |
| Typ_lax | 1 | 1 | 1 | 0 | 0 | 0 | 0 | 0 | 0 |
| Utr_vul | 1 | 0 | 1 | 1 | 0 | 1 | 0 | 0 | 0 |
| Val_spi | 1 | 0 | 1 | 0 | 0 | 0 | 1 | 0 | 0 |
| Wol_arr | 0 | 0 | 0 | 0 | 1 | 0 | 0 | 0 | 0 |

**Table S3** Strength and direction of associations between functional traits and the first two axes of the species functional trait space (PCoA).

| Trait | PCoA1 | PCoA2 |
| --- | --- | --- |
| emg | -0.983 | -0.186 |
| prnnl | -0.055 | 0.999 |
| annl | 0.055 | -0.999 |
| amph | -0.984 | -0.180 |
| anchr | -0.667 | 0.745 |
| clonlty | 0.305 | 0.952 |
| sbm | 0.734 | 0.679 |
| lftxt | -0.989 | 0.151 |
| bdyflx | 1.000 | -0.007 |
| rhzm | -0.388 | 0.922 |
| lfarea | -0.946 | 0.324 |
| sds | -0.935 | 0.353 |
| frgm | 0.999 | 0.051 |
| trns | 0.994 | 0.111 |
| strcmplx | 0.739 | 0.673 |
| bdng | 0.740 | -0.673 |
| flt | 0.740 | -0.672 |
| rtnd | 0.613 | 0.790 |
| sprs | 0.948 | -0.319 |
| tbrs | -0.321 | 0.947 |
| stln | -0.168 | 0.986 |

**Table S4** PERMANOVA results testing differences in community functional composition between Flowing and Impounded systems based on site positions in functional trait space.

| Term | Df | SumOfSqs | R^2^ | F | Pr(>F) |
| --- | --- | --- | --- | --- | --- |
| Model | 1 | 0.049 | 0.038 | 3.902 | 0.029 |
| Residual | 100 | 1.261 | 0.962 | - | - |
| Total | 101 | 1.310 | 1.000 | - | - |

**Table S5** Eigenvalues of the RLQ analysis describing joint structure between environmental variables, species composition, and functional traits.

| Axis | 1 | 2 | 3 | 4 | 5 | 6 | 7 | 8 | 9 | 10 |
| --- | --- | --- | --- | --- | --- | --- | --- | --- | --- | --- |
| Eigenvalue | 0.437 | 0.287 | 0.079 | 0.051 | 0.022 | 0.007 | 0.006 | 0.003 | 0.002 | 0.001 |

**Table S6** Results of fourth-corner analysis showing associations between environmental variables and functional traits. For each environment x trait pair (n = 210), the fourth-corner statistic (D2), unadjusted p-value, and global false discovery rate (FDR)-adjusted p-value (q-value) are reported. Directions indicate the sign of the associations. None of the associations remained significant after FDR correction. Significance codes: ns – not significant; *** - p ≤ 0.001; ** - p ≤ 0.01; * - p ≤ 0.05.

| Environmental variable | Trait | Association | p-value | q-value | Direction | p-value | q-value |
| --- | --- | --- | --- | --- | --- | --- | --- |
| cond | sbm | -0.175 | 0.001 | 0.147 | negative | *** | ns |
| w_t | amph | -0.184 | 0.004 | 0.343 | negative | ** | ns |
| cond | lfarea | 0.152 | 0.005 | 0.343 | positive | ** | ns |
| chn_w | rhzm | 0.109 | 0.010 | 0.525 | positive | ** | ns |
| w_t | emg | -0.152 | 0.020 | 0.596 | negative | * | ns |
| avg_hmrb | lfarea | 0.123 | 0.022 | 0.596 | positive | * | ns |
| clc_rchns | emg | 0.141 | 0.023 | 0.596 | positive | * | ns |
| elev | flt | -0.171 | 0.033 | 0.596 | negative | * | ns |
| elev | bdng | -0.169 | 0.033 | 0.596 | negative | * | ns |
| clc_rchns | amph | 0.131 | 0.035 | 0.596 | positive | * | ns |
| cond | emg | 0.117 | 0.037 | 0.596 | positive | * | ns |
| elev | sbm | 0.177 | 0.039 | 0.596 | positive | * | ns |
| elev | lfarea | -0.175 | 0.040 | 0.596 | negative | * | ns |
| avg_hmrb | sbm | -0.108 | 0.046 | 0.596 | negative | * | ns |
| cond | amph | 0.112 | 0.047 | 0.596 | positive | * | ns |
| clc_rchns | sds | 0.121 | 0.053 | 0.596 | positive | ns | ns |
| rip_w | rtnd | -0.091 | 0.053 | 0.596 | negative | ns | ns |
| tp | emg | 0.106 | 0.053 | 0.596 | positive | ns | ns |
| cond | bdyflx | -0.108 | 0.055 | 0.596 | negative | ns | ns |
| cond | lftxt | 0.107 | 0.060 | 0.596 | positive | ns | ns |
| chn_w | amph | -0.087 | 0.062 | 0.596 | negative | ns | ns |
| avg_hmrb | bdng | 0.095 | 0.062 | 0.596 | positive | ns | ns |
| clc_rchns | anchr | 0.113 | 0.069 | 0.615 | positive | ns | ns |
| chn_w | sbm | 0.082 | 0.070 | 0.615 | positive | ns | ns |
| bnk_slp | emg | -0.078 | 0.086 | 0.629 | negative | ns | ns |
| rip_w | tbrs | 0.077 | 0.086 | 0.629 | positive | ns | ns |
| cond | rtnd | -0.097 | 0.089 | 0.629 | negative | ns | ns |
| chn_w | emg | -0.079 | 0.090 | 0.629 | negative | ns | ns |
| din | emg | 0.083 | 0.092 | 0.629 | positive | ns | ns |
| clc_rchns | flt | -0.106 | 0.093 | 0.629 | negative | ns | ns |
| elev | rtnd | 0.137 | 0.099 | 0.629 | positive | ns | ns |
| tp | amph | 0.092 | 0.099 | 0.629 | positive | ns | ns |
| tp | sprs | -0.097 | 0.102 | 0.629 | negative | ns | ns |
| elev | stln | -0.125 | 0.104 | 0.629 | negative | ns | ns |
| avg_hmrb | frgm | -0.091 | 0.107 | 0.629 | negative | ns | ns |
| chn_w | stln | -0.071 | 0.113 | 0.629 | negative | ns | ns |
| bnk_slp | lfarea | -0.068 | 0.114 | 0.629 | negative | ns | ns |
| w_t | sds | -0.102 | 0.123 | 0.629 | negative | ns | ns |
| din | amph | 0.076 | 0.126 | 0.629 | positive | ns | ns |
| tp | lfarea | 0.086 | 0.127 | 0.629 | positive | ns | ns |
| avg_hmrb | flt | 0.081 | 0.128 | 0.629 | positive | ns | ns |
| elev | anchr | 0.129 | 0.129 | 0.629 | positive | ns | ns |
| clc_rchns | bdng | -0.096 | 0.131 | 0.629 | negative | ns | ns |
| chn_w | rtnd | -0.066 | 0.133 | 0.629 | negative | ns | ns |
| avg_hmrb | bdyflx | -0.082 | 0.138 | 0.629 | negative | ns | ns |
| avg_hmrb | emg | 0.078 | 0.144 | 0.629 | positive | ns | ns |
| w_t | clonlty | 0.099 | 0.144 | 0.629 | positive | ns | ns |
| cond | bdng | 0.079 | 0.147 | 0.629 | positive | ns | ns |
| cond | clonlty | 0.084 | 0.147 | 0.629 | positive | ns | ns |
| chn_w | sprs | -0.063 | 0.164 | 0.677 | negative | ns | ns |
| rip_w | annl | 0.067 | 0.170 | 0.677 | positive | ns | ns |
| rip_w | prnnl | -0.067 | 0.170 | 0.677 | negative | ns | ns |
| bnk_slp | amph | -0.063 | 0.171 | 0.677 | negative | ns | ns |
| cond | rhzm | 0.077 | 0.189 | 0.72 | positive | ns | ns |
| cond | frgm | -0.076 | 0.191 | 0.72 | negative | ns | ns |
| bnk_slp | sbm | 0.058 | 0.194 | 0.72 | positive | ns | ns |
| chn_w | frgm | -0.056 | 0.199 | 0.72 | negative | ns | ns |
| avg_hmrb | rtnd | -0.072 | 0.199 | 0.72 | negative | ns | ns |
| elev | bdyflx | 0.111 | 0.211 | 0.75 | positive | ns | ns |
| din | sprs | -0.060 | 0.220 | 0.771 | negative | ns | ns |
| rip_w | anchr | -0.059 | 0.229 | 0.78 | negative | ns | ns |
| clc_rchns | sprs | -0.078 | 0.231 | 0.78 | negative | ns | ns |
| bnk_slp | stln | -0.049 | 0.239 | 0.78 | negative | ns | ns |
| bnk_slp | frgm | 0.051 | 0.246 | 0.78 | positive | ns | ns |
| chn_w | strcmplx | -0.051 | 0.248 | 0.78 | negative | ns | ns |
| cond | annl | -0.066 | 0.253 | 0.78 | negative | ns | ns |
| cond | prnnl | 0.066 | 0.253 | 0.78 | positive | ns | ns |
| w_t | flt | 0.075 | 0.255 | 0.78 | positive | ns | ns |
| w_t | anchr | -0.074 | 0.258 | 0.78 | negative | ns | ns |
| clc_rchns | clonlty | -0.071 | 0.262 | 0.78 | negative | ns | ns |
| chn_w | bdng | -0.049 | 0.264 | 0.78 | negative | ns | ns |
| chn_w | tbrs | 0.052 | 0.267 | 0.78 | positive | ns | ns |
| rip_w | rhzm | -0.054 | 0.278 | 0.8 | negative | ns | ns |
| elev | sds | 0.090 | 0.291 | 0.819 | positive | ns | ns |
| elev | frgm | 0.092 | 0.299 | 0.819 | positive | ns | ns |
| elev | annl | -0.089 | 0.301 | 0.819 | negative | ns | ns |
| elev | prnnl | 0.089 | 0.301 | 0.819 | positive | ns | ns |
| din | anchr | 0.052 | 0.308 | 0.819 | positive | ns | ns |
| tp | sbm | -0.059 | 0.311 | 0.819 | negative | ns | ns |
| chn_w | sds | 0.044 | 0.312 | 0.819 | positive | ns | ns |
| din | stln | 0.041 | 0.323 | 0.837 | positive | ns | ns |
| rip_w | sbm | -0.048 | 0.331 | 0.847 | negative | ns | ns |
| avg_hmrb | tbrs | -0.053 | 0.346 | 0.875 | negative | ns | ns |
| tp | frgm | -0.056 | 0.353 | 0.883 | negative | ns | ns |
| elev | strcmplx | 0.082 | 0.361 | 0.893 | positive | ns | ns |
| rip_w | clonlty | -0.043 | 0.381 | 0.925 | negative | ns | ns |
| rip_w | flt | 0.044 | 0.383 | 0.925 | positive | ns | ns |
| avg_hmrb | amph | 0.047 | 0.393 | 0.937 | positive | ns | ns |
| avg_hmrb | strcmplx | -0.046 | 0.403 | 0.937 | negative | ns | ns |
| w_t | bdng | 0.054 | 0.404 | 0.937 | positive | ns | ns |
| avg_hmrb | clonlty | 0.047 | 0.410 | 0.937 | positive | ns | ns |
| tp | sds | 0.050 | 0.411 | 0.937 | positive | ns | ns |
| cond | tbrs | -0.038 | 0.422 | 0.946 | negative | ns | ns |
| clc_rchns | frgm | -0.051 | 0.423 | 0.946 | negative | ns | ns |
| bnk_slp | rtnd | 0.033 | 0.435 | 0.951 | positive | ns | ns |
| din | lfarea | 0.040 | 0.437 | 0.951 | positive | ns | ns |
| w_t | tbrs | 0.049 | 0.443 | 0.951 | positive | ns | ns |
| w_t | sbm | 0.049 | 0.465 | 0.951 | positive | ns | ns |
| avg_hmrb | stln | 0.037 | 0.480 | 0.951 | positive | ns | ns |
| w_t | strcmplx | -0.048 | 0.480 | 0.951 | negative | ns | ns |
| avg_hmrb | lftxt | 0.038 | 0.480 | 0.951 | positive | ns | ns |
| elev | lftxt | -0.063 | 0.484 | 0.951 | negative | ns | ns |
| tp | bdyflx | -0.040 | 0.486 | 0.951 | negative | ns | ns |
| tp | anchr | 0.040 | 0.492 | 0.951 | positive | ns | ns |
| chn_w | lftxt | 0.031 | 0.492 | 0.951 | positive | ns | ns |
| tp | rhzm | 0.039 | 0.500 | 0.951 | positive | ns | ns |
| tp | bdng | 0.031 | 0.503 | 0.951 | positive | ns | ns |
| clc_rchns | lfarea | 0.043 | 0.504 | 0.951 | positive | ns | ns |
| tp | trns | 0.032 | 0.511 | 0.951 | positive | ns | ns |
| tp | tbrs | -0.041 | 0.516 | 0.951 | negative | ns | ns |
| clc_rchns | rhzm | 0.040 | 0.518 | 0.951 | positive | ns | ns |
| rip_w | strcmplx | -0.032 | 0.522 | 0.951 | negative | ns | ns |
| avg_hmrb | sprs | -0.039 | 0.522 | 0.951 | negative | ns | ns |
| chn_w | flt | -0.028 | 0.527 | 0.951 | negative | ns | ns |
| bnk_slp | strcmplx | 0.027 | 0.536 | 0.951 | positive | ns | ns |
| bnk_slp | trns | 0.026 | 0.536 | 0.951 | positive | ns | ns |
| din | sbm | -0.032 | 0.541 | 0.951 | negative | ns | ns |
| avg_hmrb | sds | -0.030 | 0.565 | 0.951 | negative | ns | ns |
| din | rtnd | 0.026 | 0.572 | 0.951 | positive | ns | ns |
| elev | rhzm | 0.051 | 0.577 | 0.951 | positive | ns | ns |
| w_t | trns | 0.036 | 0.578 | 0.951 | positive | ns | ns |
| w_t | rtnd | -0.039 | 0.584 | 0.951 | negative | ns | ns |
| clc_rchns | stln | -0.033 | 0.585 | 0.951 | negative | ns | ns |
| din | frgm | -0.029 | 0.586 | 0.951 | negative | ns | ns |
| din | trns | 0.023 | 0.594 | 0.951 | positive | ns | ns |
| cond | strcmplx | -0.032 | 0.594 | 0.951 | negative | ns | ns |
| din | strcmplx | -0.027 | 0.596 | 0.951 | negative | ns | ns |
| din | clonlty | 0.028 | 0.597 | 0.951 | positive | ns | ns |
| din | bdng | 0.021 | 0.598 | 0.951 | positive | ns | ns |
| rip_w | emg | 0.026 | 0.608 | 0.951 | positive | ns | ns |
| clc_rchns | annl | -0.033 | 0.608 | 0.951 | negative | ns | ns |
| clc_rchns | prnnl | 0.033 | 0.608 | 0.951 | positive | ns | ns |
| cond | flt | 0.029 | 0.617 | 0.951 | positive | ns | ns |
| w_t | rhzm | 0.034 | 0.620 | 0.951 | positive | ns | ns |
| bnk_slp | bdyflx | 0.021 | 0.622 | 0.951 | positive | ns | ns |
| cond | trns | -0.026 | 0.635 | 0.951 | negative | ns | ns |
| bnk_slp | flt | -0.020 | 0.637 | 0.951 | negative | ns | ns |
| avg_hmrb | rhzm | 0.025 | 0.642 | 0.951 | positive | ns | ns |
| din | sds | 0.025 | 0.648 | 0.951 | positive | ns | ns |
| chn_w | trns | -0.019 | 0.658 | 0.951 | negative | ns | ns |
| bnk_slp | annl | -0.019 | 0.661 | 0.951 | negative | ns | ns |
| bnk_slp | prnnl | 0.019 | 0.661 | 0.951 | positive | ns | ns |
| elev | clonlty | -0.041 | 0.662 | 0.951 | negative | ns | ns |
| elev | emg | -0.039 | 0.673 | 0.951 | negative | ns | ns |
| elev | sprs | 0.036 | 0.677 | 0.951 | positive | ns | ns |
| w_t | sprs | 0.028 | 0.680 | 0.951 | positive | ns | ns |
| din | lftxt | -0.021 | 0.682 | 0.951 | negative | ns | ns |
| bnk_slp | lftxt | -0.018 | 0.686 | 0.951 | negative | ns | ns |
| bnk_slp | tbrs | -0.015 | 0.686 | 0.951 | negative | ns | ns |
| rip_w | amph | 0.020 | 0.690 | 0.951 | positive | ns | ns |
| clc_rchns | trns | -0.025 | 0.706 | 0.951 | negative | ns | ns |
| bnk_slp | sprs | -0.014 | 0.712 | 0.951 | negative | ns | ns |
| clc_rchns | bdyflx | -0.022 | 0.723 | 0.951 | negative | ns | ns |
| bnk_slp | anchr | -0.016 | 0.728 | 0.951 | negative | ns | ns |
| rip_w | bdng | 0.017 | 0.736 | 0.951 | positive | ns | ns |
| cond | anchr | 0.019 | 0.749 | 0.951 | positive | ns | ns |
| w_t | annl | 0.021 | 0.749 | 0.951 | positive | ns | ns |
| w_t | prnnl | -0.021 | 0.749 | 0.951 | negative | ns | ns |
| din | flt | -0.017 | 0.750 | 0.951 | negative | ns | ns |
| din | annl | -0.018 | 0.751 | 0.951 | negative | ns | ns |
| din | prnnl | 0.018 | 0.751 | 0.951 | positive | ns | ns |
| tp | clonlty | 0.022 | 0.752 | 0.951 | positive | ns | ns |
| din | tbrs | -0.017 | 0.760 | 0.951 | negative | ns | ns |
| cond | sds | -0.018 | 0.761 | 0.951 | negative | ns | ns |
| tp | lftxt | 0.016 | 0.768 | 0.951 | positive | ns | ns |
| elev | trns | -0.022 | 0.770 | 0.951 | negative | ns | ns |
| w_t | stln | -0.021 | 0.771 | 0.951 | negative | ns | ns |
| elev | amph | 0.025 | 0.777 | 0.951 | positive | ns | ns |
| clc_rchns | lftxt | 0.017 | 0.777 | 0.951 | positive | ns | ns |
| avg_hmrb | trns | 0.013 | 0.779 | 0.951 | positive | ns | ns |
| w_t | frgm | 0.019 | 0.779 | 0.951 | positive | ns | ns |
| bnk_slp | sds | -0.012 | 0.783 | 0.951 | negative | ns | ns |
| tp | strcmplx | 0.016 | 0.783 | 0.951 | positive | ns | ns |
| rip_w | sds | -0.012 | 0.800 | 0.955 | negative | ns | ns |
| din | bdyflx | 0.013 | 0.801 | 0.955 | positive | ns | ns |
| tp | annl | -0.017 | 0.805 | 0.955 | negative | ns | ns |
| tp | prnnl | 0.017 | 0.805 | 0.955 | positive | ns | ns |
| avg_hmrb | annl | 0.011 | 0.821 | 0.963 | positive | ns | ns |
| avg_hmrb | prnnl | -0.011 | 0.821 | 0.963 | negative | ns | ns |
| cond | sprs | -0.011 | 0.834 | 0.97 | negative | ns | ns |
| tp | flt | 0.010 | 0.836 | 0.97 | positive | ns | ns |
| cond | stln | 0.011 | 0.849 | 0.97 | positive | ns | ns |
| elev | tbrs | -0.014 | 0.852 | 0.97 | negative | ns | ns |
| chn_w | lfarea | -0.008 | 0.855 | 0.97 | negative | ns | ns |
| bnk_slp | bdng | -0.007 | 0.856 | 0.97 | negative | ns | ns |
| bnk_slp | rhzm | -0.009 | 0.859 | 0.97 | negative | ns | ns |
| clc_rchns | tbrs | 0.008 | 0.872 | 0.975 | positive | ns | ns |
| rip_w | stln | 0.009 | 0.873 | 0.975 | positive | ns | ns |
| tp | stln | 0.005 | 0.882 | 0.98 | positive | ns | ns |
| chn_w | bdyflx | -0.007 | 0.886 | 0.98 | negative | ns | ns |
| w_t | lftxt | 0.010 | 0.892 | 0.981 | positive | ns | ns |
| rip_w | trns | 0.005 | 0.910 | 0.993 | positive | ns | ns |
| avg_hmrb | anchr | 0.007 | 0.913 | 0.993 | positive | ns | ns |
| rip_w | lfarea | 0.006 | 0.918 | 0.994 | positive | ns | ns |
| din | rhzm | 0.004 | 0.937 | 0.995 | positive | ns | ns |
| w_t | bdyflx | -0.006 | 0.940 | 0.995 | negative | ns | ns |
| chn_w | annl | -0.003 | 0.941 | 0.995 | negative | ns | ns |
| chn_w | prnnl | 0.003 | 0.941 | 0.995 | positive | ns | ns |
| clc_rchns | strcmplx | 0.005 | 0.942 | 0.995 | positive | ns | ns |
| rip_w | lftxt | -0.003 | 0.954 | 0.998 | negative | ns | ns |
| rip_w | sprs | 0.002 | 0.960 | 0.998 | positive | ns | ns |
| rip_w | bdyflx | 0.002 | 0.964 | 0.998 | positive | ns | ns |
| chn_w | clonlty | -0.002 | 0.970 | 0.998 | negative | ns | ns |
| bnk_slp | clonlty | 0.001 | 0.970 | 0.998 | positive | ns | ns |
| clc_rchns | sbm | -0.001 | 0.977 | 0.998 | negative | ns | ns |
| clc_rchns | rtnd | -0.001 | 0.988 | 0.998 | negative | ns | ns |
| tp | rtnd | 0.000 | 0.990 | 0.998 | negative | ns | ns |
| chn_w | anchr | 0.000 | 0.995 | 0.998 | positive | ns | ns |
| rip_w | frgm | -0.001 | 0.997 | 0.998 | negative | ns | ns |
| w_t | lfarea | 0.000 | 0.998 | 0.998 | positive | ns | ns |

## Supplementary Figures


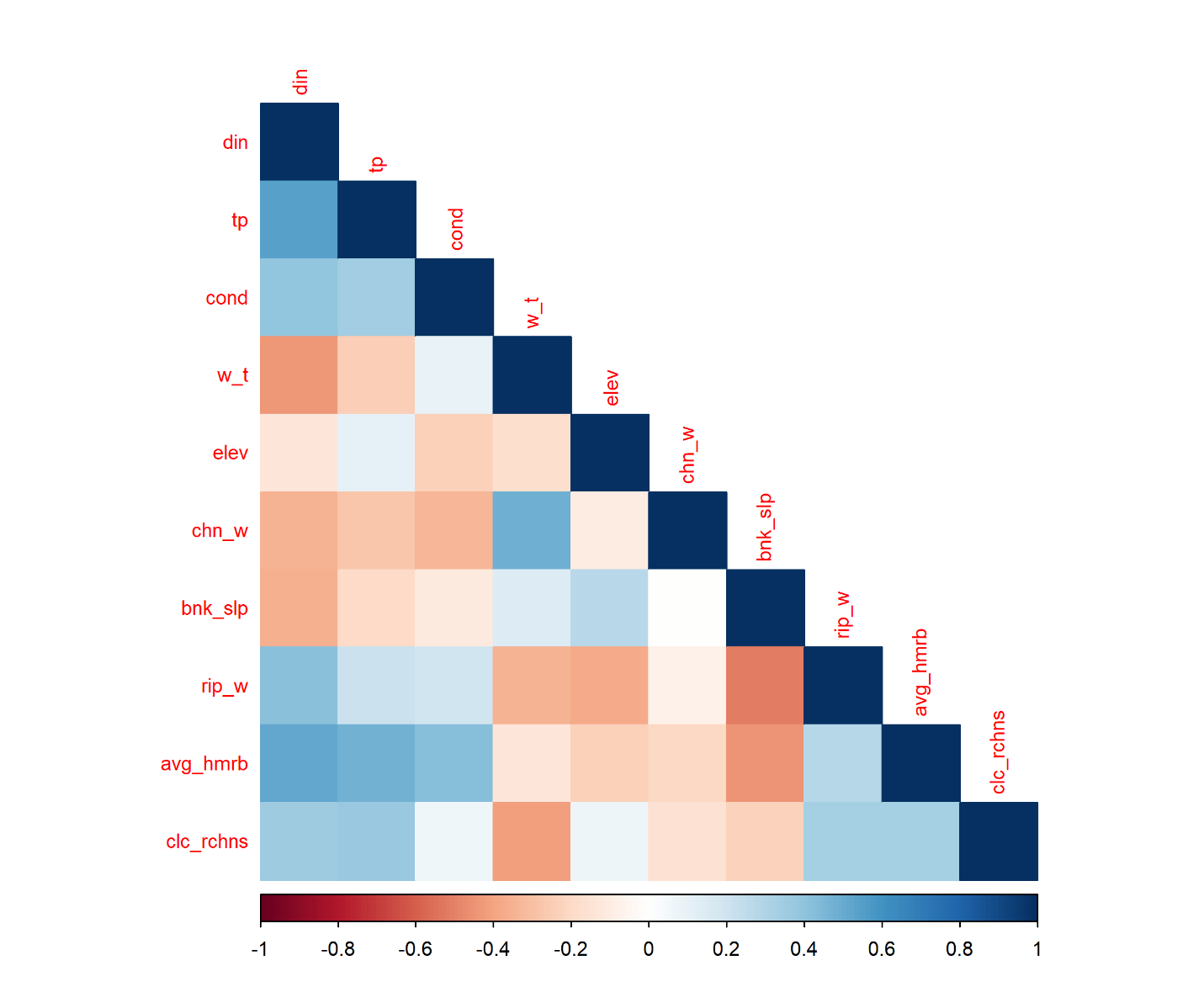


**Supplementary Figure S1** Spearman correlation matrix of environmental variables used in trait-environment analyses. Colour intensity indicates the strength and direction of correlations.


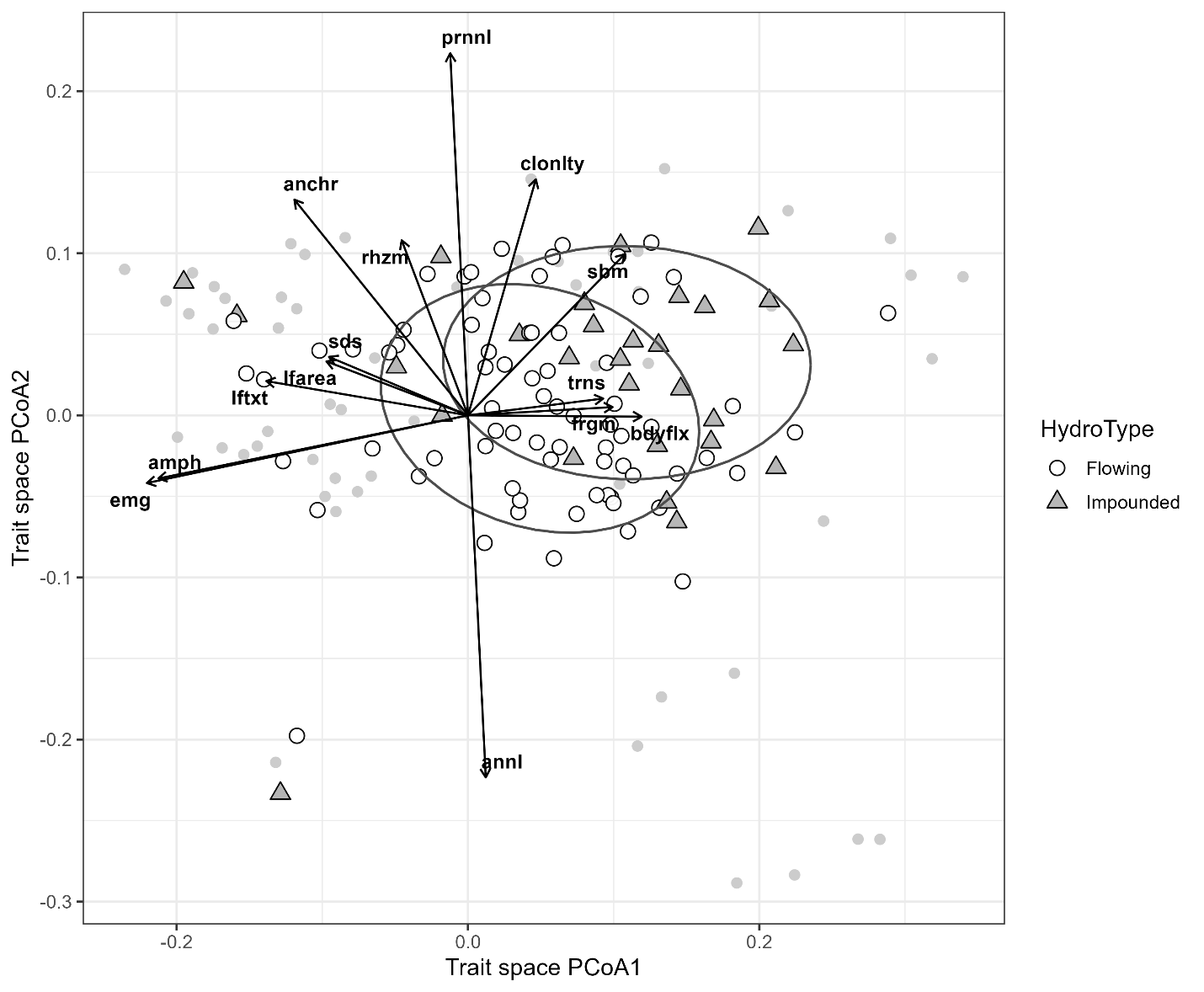


**Supplementary Figure S2** Projection of site-level macrophyte communities into the species functional trait space with fitted trait vectors overlaid. Gray points represent species, symbols indicate sites distinguished by hydrological type, and black arrows show trait vectors with *r^2^* > 0.3.


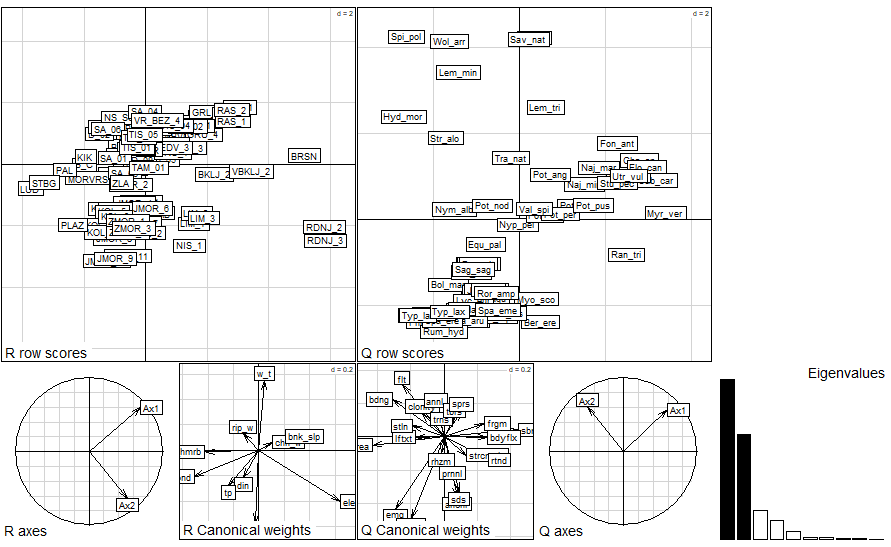


**Supplementary Figure S3.** Full RLQ ordination showing the joint structure of environmental variables, community composition, and functional traits across the first two RLQ axes, following the graphical framework of Dray et al. (2014).


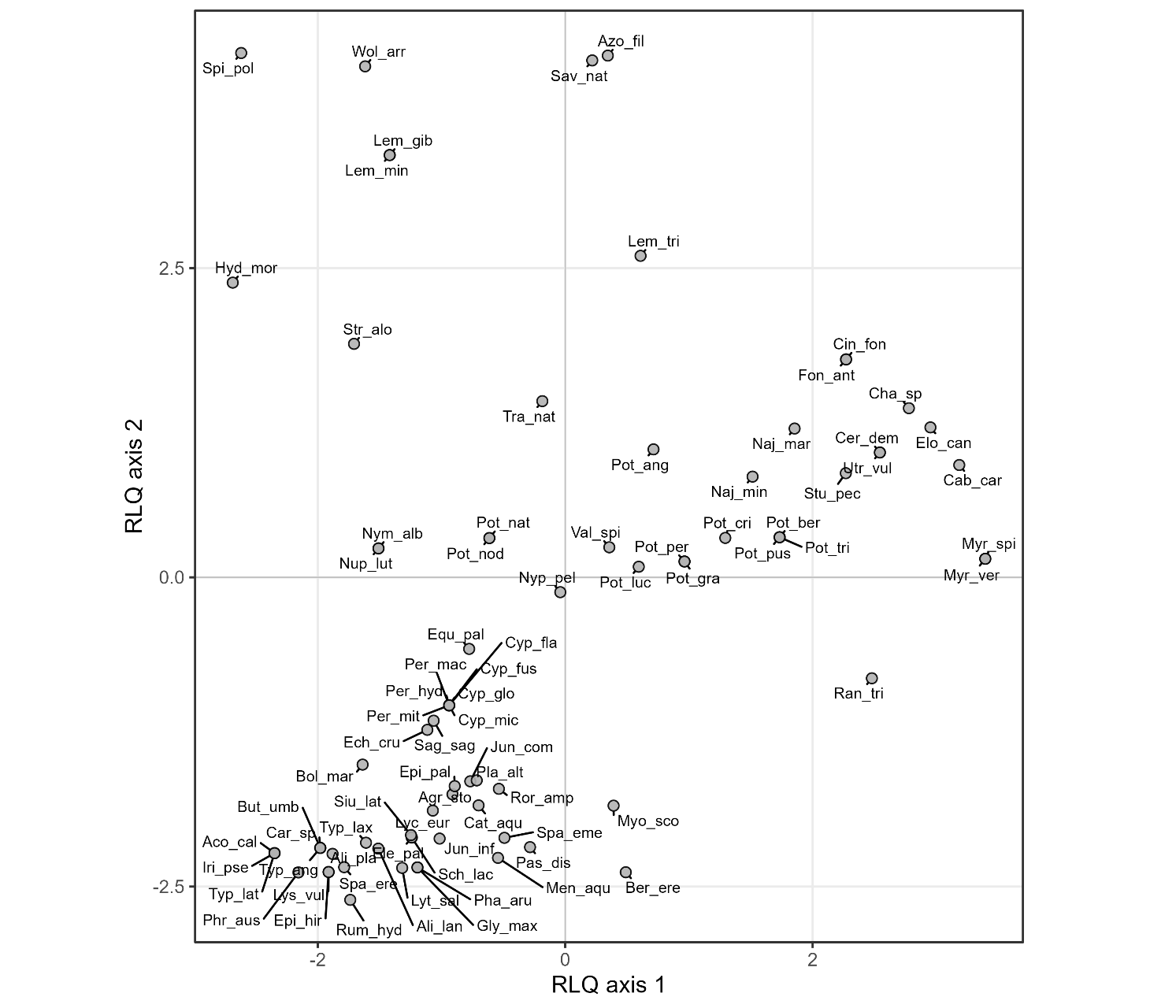


**Supplementary Figure S4.** RLQ ordination of species scores along the first two RLQ axes. The figure shows the position of macrophyte species in the joint trait–environment space defined by the RLQ analysis.


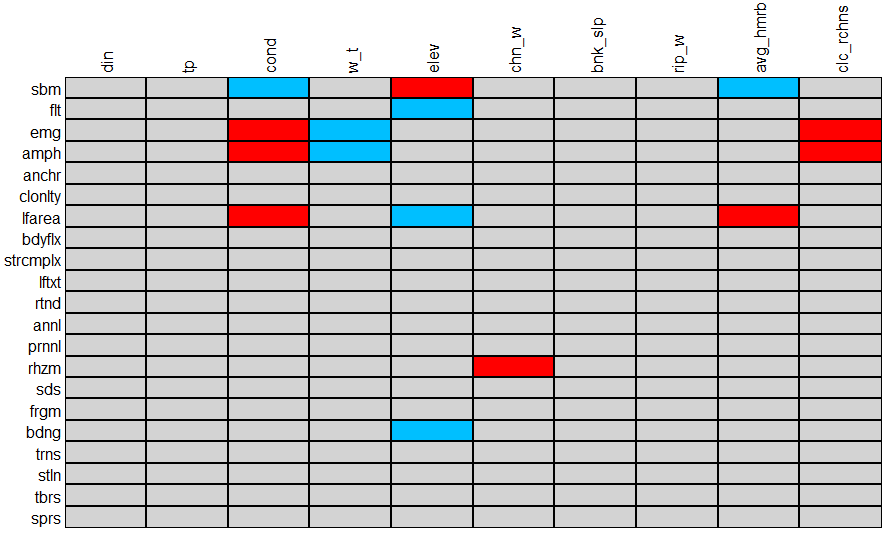


**Supplementary Figure S5** Results of fourth-corner tests, illustrating associations between environmental variables and functional traits before the correction for multiple testing. At the *α* = 0.05 level, significant positive associations are represented by red cells and negative ones by blue cells. None of the associations remained significant after correction for multiple testing using false discovery rate (FDR) adjustment.

# R script

# ============================================================

# # Inputs expected in project root:

# traits.csv, species.csv, environmental.csv, spatial.csv

#

# Outputs:

# outputs/figures, outputs/tables, outputs/rds

# ============================================================

rm(list = ls())

# ----------------------------

# 0) Packages

# ----------------------------

pkgs <- c(

"tidyverse", "janitor",

"cluster", "vegan", "corrplot",

"ggrepel", "ade4", "geosphere",

"patchwork", "viridis"

)

to_install <- pkgs[!pkgs %in% installed.packages()[, "Package"]]

if (length(to_install) > 0) install.packages(to_install, dependencies = TRUE)

invisible(lapply(pkgs, library, character.only = TRUE))

# ----------------------------

# 1) Paths & helpers

# ----------------------------

outdir <- "outputs"

dir.create(outdir, showWarnings = FALSE)

dir.create(file.path(outdir, "figures"), showWarnings = FALSE, recursive = TRUE)

dir.create(file.path(outdir, "tables"), showWarnings = FALSE, recursive = TRUE)

dir.create(file.path(outdir, "rds"), showWarnings = FALSE, recursive = TRUE)

save_csv <- function(x, fname) readr::write_csv(x, file.path(outdir, "tables", fname))

save_rds <- function(x, fname) saveRDS(x, file.path(outdir, "rds", fname))

save_fig <- function(p, fname, w = 7.5, h = 6, dpi = 400) {

ggsave(filename = file.path(outdir, "figures", fname), plot = p, width = w, height = h, dpi = dpi)

}

# ----------------------------

# 2) Read data (single, consistent read)

# ----------------------------

traits_raw <- readr::read_csv("traits.csv", show_col_types = FALSE) |> janitor::clean_names()

comm_raw <- readr::read_csv("species.csv", show_col_types = FALSE) |> janitor::clean_names()

env_raw <- readr::read_csv("environmental.csv", show_col_types = FALSE) |> janitor::clean_names()

spat_raw <- readr::read_csv("spatial.csv", show_col_types = FALSE) |> janitor::clean_names()

# Required IDs

stopifnot("site_id" %in% names(comm_raw))

stopifnot("site_id" %in% names(env_raw))

stopifnot("site_id" %in% names(spat_raw))

stopifnot("code" %in% names(traits_raw))

# Matrices

comm <- comm_raw |>

mutate(site_id = as.character(site_id)) |>

tibble::column_to_rownames("site_id") |>

as.data.frame() |>

mutate(across(everything(), as.numeric))

env <- env_raw |>

mutate(site_id = as.character(site_id)) |>

tibble::column_to_rownames("site_id") |>

as.data.frame()

spat <- spat_raw |>

mutate(site_id = as.character(site_id)) |>

tibble::column_to_rownames("site_id") |>

as.data.frame()

# HydroType: prefer env; fallback to spatial

if (!"hydro_type" %in% names(env) && "hydro_type" %in% names(spat)) {

env$hydro_type <- spat$hydro_type[match(rownames(env), rownames(spat))]

}

stopifnot("hydro_type" %in% names(env))

env$hydro_type <- factor(env$hydro_type, levels = c("Flowing", "Impounded"))

# ----------------------------

# 3) Trait schema + QC

# ----------------------------

req <- c(

"code","name",

"sbm","flt","emg",

"amph","anchr","clonlty","lfarea","bdyflx","strcmplx","lftxt","rtnd",

"annl","prnnl",

"rhzm","sds","frgm","bdng","trns","stln","tbrs","sprs"

)

missing_cols <- setdiff(req, names(traits_raw))

if (length(missing_cols) > 0) stop("Missing columns in traits.csv: ", paste(missing_cols, collapse = ", "))

trait_cols <- setdiff(req, c("code","name"))

traits <- traits_raw |>

mutate(across(all_of(trait_cols), ~as.numeric(.))) |>

mutate(code = as.character(code), name = as.character(name))

# Missingness

miss_tbl <- traits |>

summarise(across(all_of(trait_cols), ~sum(is.na(.)))) |>

pivot_longer(everything(), names_to = "trait", values_to = "n_missing") |>

arrange(desc(n_missing))

save_csv(miss_tbl, "S1_traits_missingness.csv")

# Save duplicates (informative)

save_csv(traits |> count(code) |> filter(n > 1), "S1b_trait_duplicate_codes.csv")

save_csv(traits |> count(name) |> filter(n > 1), "S1c_trait_duplicate_names.csv")

# Value checks

binary_cols <- c("sbm","flt","emg","amph","rtnd","annl","prnnl","rhzm","sds","frgm","bdng","trns","stln","tbrs","sprs")

ordinal_ranges <- list(anchr = c(0,2), clonlty = c(0,2), lfarea = c(1,4), bdyflx = c(1,3), strcmplx = c(1,3), lftxt = c(1,3))

bad_binary <- traits |>

pivot_longer(all_of(binary_cols), names_to = "trait", values_to = "value") |>

filter(!is.na(value), !value %in% c(0,1)) |>

arrange(trait, value)

save_csv(bad_binary, "S2_traits_bad_binary_values.csv")

bad_ordinal <- purrr::map_dfr(names(ordinal_ranges), function(nm) {

lo <- ordinal_ranges[[nm]][1]; hi <- ordinal_ranges[[nm]][2]

traits |>

filter(!is.na(.data[[nm]]), .data[[nm]] < lo | .data[[nm]] > hi) |>

transmute(code, name, trait = nm, value = .data[[nm]], lo, hi)

})

save_csv(bad_ordinal, "S3_traits_bad_ordinal_values.csv")

# Exclude zero-variance traits before ordination

trait_var <- traits |>

summarise(across(all_of(trait_cols), ~sd(.x, na.rm = TRUE))) |>

pivot_longer(everything(), names_to = "trait", values_to = "sd") |>

mutate(zero_var = is.na(sd) | sd == 0)

save_csv(trait_var, "S4_traits_sd.csv")

trait_cols_use <- trait_var |> filter(!zero_var) |> pull(trait)

# ----------------------------

# 4) Harmonize species between comm and traits

# ----------------------------

sp_keep <- intersect(colnames(comm), traits$code)

if (length(sp_keep) < 10) stop("Very few shared species between species.csv and traits.csv. Check species codes.")

comm2 <- comm[, sp_keep, drop = FALSE]

sites_keep <- Reduce(intersect, list(rownames(comm2), rownames(env), rownames(spat)))

comm2 <- comm2[sites_keep, , drop = FALSE]

env2 <- env[sites_keep, , drop = FALSE]

spat2 <- spat[sites_keep, , drop = FALSE]

# ----------------------------

# 5) Trait PCoA (Gower) + envfit vectors (descriptive)

# ----------------------------

traits_mat <- traits |>

filter(code %in% sp_keep) |>

select(all_of(trait_cols_use))

gower_dist <- cluster::daisy(traits_mat, metric = "gower")

pcoa <- cmdscale(as.dist(gower_dist), k = 2, eig = TRUE)

scores_sp <- as.data.frame(pcoa$points)

names(scores_sp) <- c("Axis1", "Axis2")

scores_sp$Code <- traits$code[match(rownames(traits_mat), traits$code)]

scores_sp$Name <- traits$name[match(rownames(traits_mat), traits$code)]

# envfit on traits (permutations = 0 => descriptive)

fit <- vegan::envfit(scores_sp[, c("Axis1","Axis2")], traits_mat, permutations = 0)

vec <- as.data.frame(fit$vectors$arrows)

vec$trait <- rownames(vec)

vec$r2 <- fit$vectors$r

mult <- 0.8 * max(abs(scores_sp$Axis1), abs(scores_sp$Axis2))

vec$xend <- vec$Axis1 * vec$r2 * mult

vec$yend <- vec$Axis2 * vec$r2 * mult

save_csv(vec |> arrange(desc(r2)), "S6_envfit_trait_vectors_full.csv")

# Filter for plotting (tune threshold if you like)

vec2 <- vec |> dplyr::filter(r2 > 0.30)

save_csv(vec2 |> arrange(desc(r2)), "S7_envfit_trait_vectors_filtered_r2gt0.3.csv")

# ----------------------------

# 6) Project sites into trait PCoA space (community-weighted position)

# ----------------------------

sp_mat <- scores_sp |>

select(Code, Axis1, Axis2) |>

tibble::column_to_rownames("Code") |>

as.matrix()

sp_mat <- sp_mat[sp_keep, , drop = FALSE]

rs <- rowSums(comm2, na.rm = TRUE)

comm_rel <- sweep(comm2, 1, rs, "/")

comm_rel[!is.finite(as.matrix(comm_rel))] <- NA

site_xy <- as.matrix(comm_rel) %*% sp_mat

site_scores <- data.frame(

SiteID = rownames(comm2),

Axis1 = site_xy[, 1],

Axis2 = site_xy[, 2],

HydroType = env2$hydro_type,

stringsAsFactors = FALSE

) |>

filter(!is.na(Axis1), !is.na(Axis2), !is.na(HydroType)) |>

mutate(HydroType = factor(HydroType, levels = c("Flowing", "Impounded")))

save_csv(site_scores, "T1_site_positions_in_trait_space.csv")

# ----------------------------

# 7) PERMANOVA (functional composition)

# ----------------------------

dist_fun2d <- dist(site_scores[, c("Axis1", "Axis2")])

perm <- vegan::adonis2(dist_fun2d ~ HydroType, data = site_scores, permutations = 9999)

perm_tbl <- as.data.frame(perm)

save_csv(tibble::rownames_to_column(perm_tbl, "Term"), "T2_PERMANOVA_trait_space_HydroType.csv")

# ----------------------------

# 8) Distance–decay (taxonomic vs functional), requires lat/long

# ----------------------------

if (all(c("latitude","longitude") %in% names(spat2))) {

coords <- spat2[match(site_scores$SiteID, rownames(spat2)), c("latitude","longitude")] |> as.matrix()

geo_km <- as.matrix(geosphere::distm(coords, fun = geosphere::distHaversine)) / 1000

rownames(geo_km) <- colnames(geo_km) <- site_scores$SiteID

comm_hel <- vegan::decostand(comm2[site_scores$SiteID, , drop = FALSE], method = "hellinger")

tax_dist <- vegan::vegdist(comm_hel, method = "bray")

fun_dist <- dist_fun2d

pair_df <- function(mat, value_name) {

idx <- which(upper.tri(mat), arr.ind = TRUE)

tibble(

site1 = rownames(mat)[idx[, 1]],

site2 = colnames(mat)[idx[, 2]],

!!value_name := mat[idx]

)

}

dd <- pair_df(geo_km, "geo_km") |>

left_join(pair_df(as.matrix(tax_dist), "tax_dissim"), by = c("site1","site2")) |>

left_join(pair_df(as.matrix(fun_dist), "fun_dissim"), by = c("site1","site2")) |>

mutate(

tax_sim = 1 - tax_dissim,

fun_sim = 1 - (fun_dissim / max(fun_dissim, na.rm = TRUE))

)

ht <- site_scores |> select(SiteID, HydroType)

dd <- dd |>

left_join(ht, by = c("site1" = "SiteID")) |> rename(ht1 = HydroType) |>

left_join(ht, by = c("site2" = "SiteID")) |> rename(ht2 = HydroType) |>

mutate(pair_type = case_when(

ht1 == "Flowing" & ht2 == "Flowing" ~ "Flowing–Flowing",

ht1 == "Impounded" & ht2 == "Impounded" ~ "Impounded–Impounded",

TRUE ~ "Flowing-Impounded"

)) |>

mutate(pair_type = factor(pair_type, levels = c("Flowing–Flowing","Impounded–Impounded","Flowing-Impounded")))

save_csv(dd, "S9_distance_decay_pairwise_table.csv")

} else {

message("NOTE: spatial.csv lacks latitude/longitude; skipping distance-decay.")

dd <- NULL

}

# ----------------------------

# 9) RLQ + fourth-corner (clean alignment, no duplicate blocks)

# ----------------------------

# Choose numeric environmental predictors (edit as needed)

needed_env <- c("din","tp","cond","w_t","elev","chn_w","bnk_slp","rip_w","avg_hmrb","clc_rchns")

R_num <- env2 |> select(any_of(needed_env)) |> select(where(is.numeric))

if (ncol(R_num) >= 2) {

R_std <- as.data.frame(scale(R_num))

L <- vegan::decostand(comm2[site_scores$SiteID, , drop = FALSE], method = "hellinger") |> as.data.frame()

Q <- traits |>

filter(code %in% colnames(L)) |>

select(code, all_of(trait_cols_use)) |>

arrange(match(code, colnames(L))) |>

tibble::column_to_rownames("code") |>

as.data.frame()

# Strict alignment

R_std <- R_std[rownames(L), , drop = FALSE]

stopifnot(identical(rownames(R_std), rownames(L)))

stopifnot(identical(rownames(Q), colnames(L)))

dL <- ade4::dudi.coa(L, scannf = FALSE, nf = 2)

dR <- ade4::dudi.pca(R_std, row.w = dL$lw, scannf = FALSE, nf = 2)

dQ <- ade4::dudi.hillsmith(Q, row.w = dL$cw, scannf = FALSE, nf = 2)

rlq_res <- ade4::rlq(dR, dL, dQ, scannf = FALSE, nf = 2)

fc <- ade4::fourthcorner(R_std, L, Q, modeltype = 6, nrepet = 9999)

fc1 <- ade4::fourthcorner(R_std, L, Q, modeltype = 6,

p.adjust.method.G = "none", p.adjust.method.D = "none",

nrepet = 9999)

fc1.adj <- ade4::p.adjust.4thcorner(fc1, p.adjust.method.G = "fdr", p.adjust.method.D = "fdr", p.adjust.D = "global")

save_rds(rlq_res, "rlq_result.rds")

save_rds(fc, "fourthcorner_result.rds")

save_rds(fc1.adj, "fourthcorner_fdr_global.rds")

rlq_eig <- tibble(axis = seq_along(rlq_res$eig), eigenvalue = rlq_res$eig)

save_csv(rlq_eig, "S10_RLQ_eigenvalues.csv")

} else {

message("NOTE: Too few numeric environmental variables for RLQ/fourth-corner. Skipping.")

}

# ----------------------------

# 10) Save core objects + session info

# ----------------------------

core <- list(

traits = traits,

traits_mat = traits_mat,

gower_dist = gower_dist,

pcoa = pcoa,

scores_species = scores_sp,

envfit = fit,

envfit_vectors = vec,

envfit_vectors_filtered = vec2,

site_scores = site_scores,

env = env2,

comm = comm2,

dd = dd

)

save_rds(core, "core_objects.rds")

sink(file.path(outdir, "tables", "S11_sessionInfo.txt"))

print(sessionInfo())

sink()

message("DONE. Outputs saved in: ", outdir)
